# *Camellia sinensis*-synthesized silver nanoparticles and meropenem combination against extensively drug-resistant *Klebsiella pneumoniae*

**DOI:** 10.1038/s41598-026-38375-0

**Published:** 2026-02-20

**Authors:** Eman M. Elmasry, Essraa Hegazy, Ghadir S. El-Housseiny, Khaled M. Aboshanab

**Affiliations:** 1 National Cancer Institute, Fom El-Khalig, Cairo University, Cairo, 11796 Egypt; 2 Department of Medical Microbiology and Immunology, Faculty of Medicine, Cairo University, Cairo, Egypt; 3 Department of Microbiology and Immunology, Faculty of Pharmacy, Ain Shams University, Cairo, 11566 Egypt

**Keywords:** Klebsiella pneumoniae, Silver nanoparticles, Meropenem, Antimicrobial resistance, Synergism, Biotechnology, Drug discovery, Microbiology

## Abstract

New strategies against antimicrobial resistance are urgently needed to address the challenges and health consequences posed by extensively drug-resistant (XDR) clinical isolates. This study explored how effective the green-synthesized silver nanoparticles alone or in combination with meropenem are in combating XDR *Klebsiella pneumoniae*. *Klebsiella pneumoniae* (*K. pneumoniae*) isolates were collected from several clinical labs and identified. After carrying out antimicrobial susceptibility testing, XDR isolates were selected for genotypic analysis using ERIC-PCR, and antibiotic resistance genes were identified. Silver nanoparticles (AgNPs) were synthesized using a *Camellia sinensis* (green tea) extract and characterized using UV-Vis spectroscopy, transmission electron microscopy (TEM), and X-ray diffraction (XRD). Particle size analysis was conducted to determine the physicochemical properties of the synthesized AgNPs. The AgNPs antibacterial effect alone or in combination with meropenem was carried out using the agar-well diffusion method, and the minimum inhibitory concentration (MIC), multiple antibiotic resistance (MAR) index and fractional inhibitory concentration index (FICI) were determined. Of the collected isolates, 67 were identified as *K. pneumoniae*, and 92.5% were considered XDR. Results showed that 10 (14.9%) and 57 (85.1%) isolates exhibited MAR index from 0.58 to 0.74, and 0.79-1.00, respectively. Upon genotypic analysis, 29 isolates were selected for further studies based on their unique or significant clustering patterns. The synthesized AgNPs exhibited strong antimicrobial activity against XDR *K. pneumoniae* strains, with inhibition zones ranging from 10 mm to 25 mm. The MIC of AgNPs ranged from 336.17 to 672.35 µg/mL. The fractional inhibitory concentration index (FICI) proved enhanced antimicrobial activity with partial (34.5%) and complete synergistic (65.5%) effects in the tested 29 nonclonal clinical isolates when AgNPs were combined with meropenem. In conclusion, a combination of AgNPs with meropenem is a useful alternative approach in combating XDR *K. pneumoniae*. Further studies are recommended for the use of this approach in clinical settings based on its benefits to enhance treatment outcomes.

**Supplementary Information:**

The online version contains supplementary material available at 10.1038/s41598-026-38375-0.

## Introduction

*Klebsiella pneumoniae*, a Gram-negative bacterium, causes serious infections, especially in immunocompromised patients, including pneumonia, urinary tract infections, meningitis, and bloodstream infections^1,2^. It is one of the main causes of nosocomial infections globally, and in Egypt, it was ranked third among healthcare-associated infections (HAIs) in 2021, accounting for 21.5% of all HAIs^3^. The strains of *K. pneumoniae* can be divided into two groups according to their accessory genome: classical strains (CKP) and hypervirulent strains (HvKP). While classical strains are opportunistic pathogens that cause pneumonia, urinary tract infection, wound infection, or bloodstream infection, HvKP strains can cause pyogenic liver abscesses, meningitis, necrotizing fasciitis, and endophthalmitis not only in immunocompromised patients but also in healthy ones^4,5^. Moreover, HvKP strains have a large virulence-related gene-carrying plasmid that might serve as an indicator for the presence of HvKP associated with more severe infection and higher antibiotic resistance^6^.

Both CKP and HvKP can form biofilms, which play an important role in bacterial virulence. It protects the bacteria from different environmental conditions, leading to the formation of invasive infections, and acts as a barrier to antibiotic action^7^. Moreover, biofilm formation enhances the horizontal gene transfer of mobile genetic elements that carry resistant genes such as ESBL (extended-spectrum beta-lactamase) and CRE (carbapenem-resistant Enterobacteriaceae) genes^8,9^. The rise of multidrug resistance (MDR) and hypervirulent strains leads to increased health costs and mortalities, making *K. pneumoniae* extensively drug-resistant (XDR) and complicating its treatment^5,10^. The XDR *K. pneumoniae* clinical isolates are a big challenge to be overcome through new antibiotics, combination therapy, antimicrobial stewardship programs, and infection control practices^11–13^. Several therapeutic options, like colistin and tigecycline in combination with meropenem, have been tried for carbapenem-resistant *K. pneumoniae*^14^. New antibiotics such as ceftazidime-avibactam, cefiderocol, and meropenem/vaborbactam showed their efficacy against MDR strains in complicated infections^15^.

However, the optimal antibiotic combination regimen to control and resolve such infections has not been established yet^10^. Moreover, developing new antibiotics is very difficult, expensive, time-consuming, and most of the time unsuccessful^16^. Hence, alternative approaches, including phage therapy, antimicrobial peptides, nanoparticles, and vaccine development, have been considered in recent studies^8,17,18^. Nanotechnology has been proposed as one potential solution to antibiotic resistance in bacteria^19^. Metal nanoparticles have broad-spectrum antimicrobial activity through different mechanisms, which leads to the death of MDR strains. The most used antimicrobial metal nanoparticles, used with or without antibiotics in combating bacterial resistance, include silver, gold, copper, and platinum. Among the commonly used metal nanoparticles, AgNPs have shown excellent effects on MDR bacterial isolates^8^. Green tea (Camellia sinensis) extract was selected for the synthesis of AgNPs due to its high content of polyphenols and proteins, which act as natural reducing and stabilizing agents. Additionally, Camellia sinensis possesses inherent antioxidant and antimicrobial properties, which can enhance the biological activity of the resulting nanoparticles. Its safety, non-toxicity, widespread availability, and eco-friendly nature make it particularly suitable for green synthesis^20,21^. Accordingly, this study aimed to evaluate the antimicrobial activity of green-synthesized AgNPs using a green tea extract either alone or in combination with meropenem against XDR *K. pneumoniae* clinical isolates.

## Materials and methods

### Collection and identification of the clinical isolates

One hundred isolates of various Gram-negative bacteria were recovered from various clinical specimens obtained from one hundred patients in the Microbiology lab at El-Demerdash Hospital. The purified Gram-negative bacteria were cultured on MacConkey agar plates and incubated overnight at 37 °C, and the characteristics of the colonies were recorded. Gram staining was performed on freshly isolated pure colonies of the test isolates to determine their morphological and microscopic characteristics, which were then noted. Besides, biochemical tests, like the triple sugar iron test, citrate utilization test, lysine decarboxylase test, rapid urease test, motility, indole, and ornithine medium test, were done for each of the isolates, and biochemical features were recorded. For definitive identification of K. *pneumoniae*, isolates were subjected to the VITEK 2 COMPACT system (bioMérieux, France). According to the manufacturer’s instructions, bacterial suspensions were inoculated onto both antimicrobial susceptibility testing (AST-N) cards and Gram-negative identification (GN) cards for analysis. A string test was done to detect hypervirulent strains as follows: The strains were inoculated onto MacConkey agar media and incubated at 37 °C for 20–24 h. The colonies on the agar media were pulled up using a loop. A positive string test result was defined as a viscous string formation of > 5 mm, indicating a hypervirulent strain^22^. All isolates were stored at 4 °C on MacConkey agar slants for short-term preservation. For long-term storage, isolates were maintained in trypticase soy broth (TSB) containing 50% glycerol at − 80 °C^23^. The study was approved by the Faculty of Pharmacy, Ain Shams University Research Ethics Committee (ACUC-FP-ASU RHDIRB2020110301, REC# 402), with the waiving of the patient consent since there was no patient contact and the isolates were obtained from unidentified clinical specimens discharged from the general microbiology diagnostic laboratory of the hospital. All methods were performed in accordance with the Declaration of Helsinki. https://www.wma.net/policies-post/wma-declaration-of-helsinki/.

### Antimicrobial susceptibility testing

The antimicrobial susceptibility tests using the Kirby-Bauer disk diffusion technique were performed according to the Clinical Laboratory Standards Institute (CLSI 2021)^24^, and the MDR phenotypes were determined as previously reported^25^. The Mueller-Hinton agar was used for susceptibility testing using the following antimicrobials: The antibiotic discs that were purchased from Biomaxima (Poland) included amoxicillin/clavulanate (AMC) (20/10 µg), ceftriaxone (CRO) (30 µg), imipenem (IMP) (10 µg), meropenem (MEM) (10 µg), chloramphenicol (C) (30 µg), tigecycline (TGC) (15 µg), and doxycycline (DO) (30 µg). The antibiotic discs that were purchased from Bioanalyse (Turkey) included piperacillin/tazobactam (TPZ) (100/10 µg), ceftazidime (CAZ) (30 µg), cefotaxime (CTX) (30 µg), cefepime (FEB) (30 µg), aztreonam (ATM) (10 µg), tobramycin (TOP) (10 µg), amikacin (AK) (30 µg), ciprofloxacin (CIP) (5 µg), levofloxacin (LEV) (5 µg), and trimethoprim/sulfamethoxazole (SXT) (2.25/23.75 µg). Gemifloxacin (GEM) (5 µg) and colistin (CL) (10 µg) antibiotic discs were purchased from Himedia (India). The MDR and XDR phenotypes were assessed as formerly described by Magiorakos et al.^25^. The multiple antibiotic resistance (MAR) index was calculated as previously reported^26^.

### Genotypic analysis

#### Genetic diversity using ERIC -PCR

The genomic DNA of the collected MDR isolates, incubated overnight in LB broth at 37 °C at 200 RPM, was extracted using the genomic DNA extraction and purification kit (Qiagen, Germany) according to the manufacturer’s instructions. The genotyping of the MDR isolates was performed by Enterobacterial Repetitive Intergenic Consensus Polymerase Chain Reaction (ERIC-PCR) using a pair of published primers. The ERIC primer sequence used was Forward: 5`-ATGTAAGCTCCTGGGGATTCA C-3` and Reverse: 5`-AAGTAAGTG ACTGGGGTGAGC G-3`, producing variable amplification products^27^. The ERIC primers were purchased from Metabion Co. (Planegg, Germany). The master mix was prepared according to EmeraldAmp GT PCR master mix (Takara Bio Inc., Japan), Code No. RR310A kit instructions. A total volume of 25 µL consisted of Emerald Amp GT PCR master mix (2x premix): 12.5 µL, PCR grade water: 5.5 µL, forward primer (20 pmol): 1 µL, reverse primer (20 pmol): 1 µL, and template DNA: 5 µL (50 nmol). The amplified products of ERIC-PCR were separated using agarose gel electrophoresis (1% agarose gel) and stained with ethidium bromide using UV radiation for visualization of the bands. The ERIC-PCR fingerprint data were then analyzed using the PyElph 1.4 software (BMC Bioinformatics USA) using a threshold of 7 for profiles and the unweighted pair group method with arithmetic average (UPGMA) method for clustering and generating dendrograms. For each dendrogram, the similarity index was calculated as follows^28^:

1 $${\mathrm{S}}\,=\,{\mathrm{1}} - {\text{ }}\left( {{\mathrm{d}}/{\mathrm{dmax}}} \right)*{\mathrm{1}}00$$

Where S = similarity index percentage, d = the distance of an isolate in the dendrogram, and dmax = the maximum distance in the dendrogram.

### Detection of antibiotic resistance genes

The study targeted several resistance genes, primers used, and their sources^29–34^ are displayed in Table S1. The PCR cycling conditions for all genes detected and for ERIC PCR previously included an initial denaturation at 94 °C for 5 min, followed by 35 cycles of denaturation at 94 °C for 30 s, specific annealing temperatures ranging from 52 °C to 62.5 °C for 30–40 s, and extension at 72 °C for 30–45 s. The final extension lasted 7–12 min, depending on the gene. Then, the DNA molecular weight marker (GeneRuler 100 bp DNA Ladder, Cat. No. SM0243, Fermentas, USA), containing 10 bands with a size range of 100–1000 bp, was used, and gel electrophoresis was done as mentioned above. For the antibiotic-resistant genes detected, the data were analyzed using SPSS Software Version 19. The data was summarized, and the chi-square test was used for comparison of the obtained data (p-value < 0.05 was significant).

### Green synthesis of silver nanoparticles (AgNPs)

Silver nanoparticles were synthesized following the method of Ali et al. with minor modifications^30^. A green tea filter bag was purchased from the local market. In a 500 mL beaker, 20 g of dried green tea powder was mixed with 100 mL of deionized water and heated at 60 °C for 10 min with gentle stirring. The mixture was then cooled to room temperature and filtered to obtain a clear green tea extract with an approximately neutral pH 7.7. For the synthesis, 15 mL of this extract was added to 100 mL of 0.0125 M AgNO₃ solution under gentle stirring. The formation of silver nanoparticles was indicated by a visible color change of the solution^35^.

### Characterization of silver nanoparticles (AgNPs)

Several techniques were used to determine AgNPs’ physicochemical properties; these techniques included: (i) A transmission electron microscope (TEM) (JEO model JEM 2100 F) was used to image the nanoparticles and to investigate the micrograph of obtained samples under an operating voltage of 160 kV. The TEM-captured images were analyzed using ImageJ software, and the collected data from ImageJ software was interpreted using Origin software for the determination of particle size and surface area. From the calculated particle size, the mass concentration was determined using several equations^36^.

2 $${\text{N total }}/{\text{ N p}}\,=\,{\text{M total }}/{\text{ M p}}$$

Where N total is equal to the total number of silver atoms in silver nitrate, N p is equal to the number of atoms in one NP, M total is equal to the total mass of AgNPs, and M p is equal to the mass of a single nanoparticle.

First, M_p_, N_p_, and N total were calculated using the following equations (Eqs. 3, 4, and 5).

3 $${\text{M p}}\,=\,\pi \rho {{\mathrm{D}}^3}/{\mathrm{6}}$$

4 $${\text{N p}}\,=\,\pi \rho {\mathrm{D3}}/{\text{6M }} \times {\text{ NA}}$$

Where ρ = density of silver (10.49 g/cm^3^), D = calculated particle size in cm, M = molecular weight of silver (107.87 amu), and NA = Avogadro’s constant (6.02 × 10^23^).

5 $${\text{N total}}\,=\,{\text{EquationNumber of moles of silver }} \times {\text{ volume of deionized water in liters }} \times {\text{ NA}}$$

Finally, the total mass of AgNPs (Mtotal) and the total mass concentration of AgNPs in one mL were calculated.

(ii) A UV-VIS spectrophotometer (Perkin Elmer, Lambda 35) was employed to identify the optical properties of AgNPs. The AgNPs solution was scanned from 300 to 800 nm, and the collected data were interpreted using Origin software for the determination of the surface plasmon resonance (SPR) of the spectra.
(iii) X-ray diffraction (XRD) using the Bruker D8 phase diffractometer, which was used to determine the phase of AgNPs in the range 10° ≤ 2θ ≤ 90° at λ = 1.5406 Å. After the determination of the phase and peak patterns, the grain size was calculated using Scherrer’s equation (Eq. 6).

6 $${\mathrm{D}}\,=\,{\mathrm{k}}.{\mathrm{l}}/\beta {\mathrm{cos}}(\theta )$$

Where D is considered the average grain size, K = constant (0.89), λ = wavelength in nm (0.15406 nm), Ɵ = diffraction angle (º), and β = full width at half maximum (FWHM) of the peak diffraction in radians^37^.

### Determination of antimicrobial activity

The antimicrobial activity of green tea-synthesized AgNPs was evaluated both qualitatively and quantitatively. For qualitative analysis, the agar well diffusion method was performed using tryptic soy agar plates surface inoculated with MDR *K. pneumoniae* isolates^8^. Each plate was divided into four quarters. In the first half, two wells were cut using a sterile borer (4–8 mm in diameter); one well was filled with AgNPs solution, and the other well was filled with green tea extract solution, which served as a control. This setup was intended to assess the inhibitory effect of AgNPs alone. In the second half, a meropenem disc was placed in one quarter, while in the last quarter, a meropenem disc that had been immersed in an AgNPs solution was placed. This arrangement was designed to evaluate the potential synergistic interaction between AgNPs and meropenem. Meropenem was used in this study in combination with AgNPs to assess whether the nanoparticles could enhance the antibacterial activity of this antibiotic against MDR *Klebsiella pneumoniae* isolates. All plates were incubated at 37 °C for 24 h. Then the inhibition zones were measured, and the results were interpreted.

For quantitative analysis, the checkerboard assay was used to determine the minimum inhibitory concentration (MIC) of AgNPs and meropenem, each alone, and to determine the synergistic effect of both using the 96-well microtiter plate dilution method^38^. Serial dilutions of AgNPs and meropenem were prepared in nutrient broth. AgNPs were twofold diluted along the columns of the microtiter plates, and meropenem was diluted along the rows. Positive and negative controls were created in columns 11 and 12, respectively. The positive control consisted of 200 µL of nutrient broth as well as the bacterial suspension, whereas the negative control had only 200 µL of nutrient broth. Subsequently, 20 µL of the bacterial culture (1 × 10^8^ CFU/mL) was added to all wells, excluding the negative control wells, and the microtiter plate was incubated for 24 h at 37 °C. After incubation, the minimum inhibitory concentration (MIC) of AgNPs, meropenem, and the combination was recorded for each isolate. The calculation of the fractional inhibitory concentration index (FICI) was performed using the following equations^39^:

7 $${\mathrm{FICI}}\,=\,{\text{FIC of AgNPs}}\,+\,{\text{FIC of Meropenem}}$$

FIC of AgNPs = MIC of AgNPs in combination with meropenem/MIC of AgNPs alone. FIC of Meropenem = MIC of meropenem in combination with AgNPs/MIC of meropenem alone.

The interpretation of the FIC index was as follows (according to the American Society of Microbiology (ASM) and Khalil et al.^38^. FICI ≤ 0.5 indicates synergy; 0.5 < FICI < 1 indicates partial synergy; FICI = 1 indicates an additive effect; 2 ≤ FICI < 4 indicates an indifferent effect; and FICI > 4 indicates antagonism.

### Statistical analysis

For the antibiotic-resistant genes detected, the data were analyzed using SPSS Software Version 19. The data was summarized, and the chi-square test was used for comparison of the obtained data (p-value < 0.05 was significant). For TEM particle size and inhibition zones for meropenem, silver nanoparticles (AgNPs), and their combination, results were recorded as mean of triplicate values ± standard deviation, and range was also included.

## Results

### Identification of the collected clinical isolates

Microscopically, a total of 100 isolates were short Gram-negative rods as examined by Gram staining. Of these, 67 strains were proven to be *K. pneumoniae*, while the rest were *E. coli*,* Enterobacter*, and other species of *Klebsiella* according to culture and biochemical analysis. On MacConkey agar, *K. Pneumoniae* isolates showed large mucoid colonies with pink pigment diffusion. Results of biochemical reactions were as follows: citrate-positive, urease-positive, lysine-positive, non-motile, ornithine-positive, indole-negative, and acid/acid in the triple sugar test result. According to the VITEK test results, all 67 clinical isolates were consistently identified as *K. pneumoniae* with a high confidence level. Of these, 10 isolates exhibited a 98% probability of accurate identification, 14 isolates a 96% probability, and the remaining isolates a 99% probability. According to the string test, *K. Pneumoniae* isolates ranged from classical to hypervirulent strains. A total of 16 isolates were identified as classical strains, while the remaining isolates (*n* = 51) exhibited viscous string formation ranging between 6 and 7 cm, indicating a hypervirulent phenotype. These results proved that hypervirulent strains (*n* = 51; 76%) are more prevalent among the collected *K. pneumoniae* isolates.

### Antimicrobial susceptibility

**Fig. 1:**
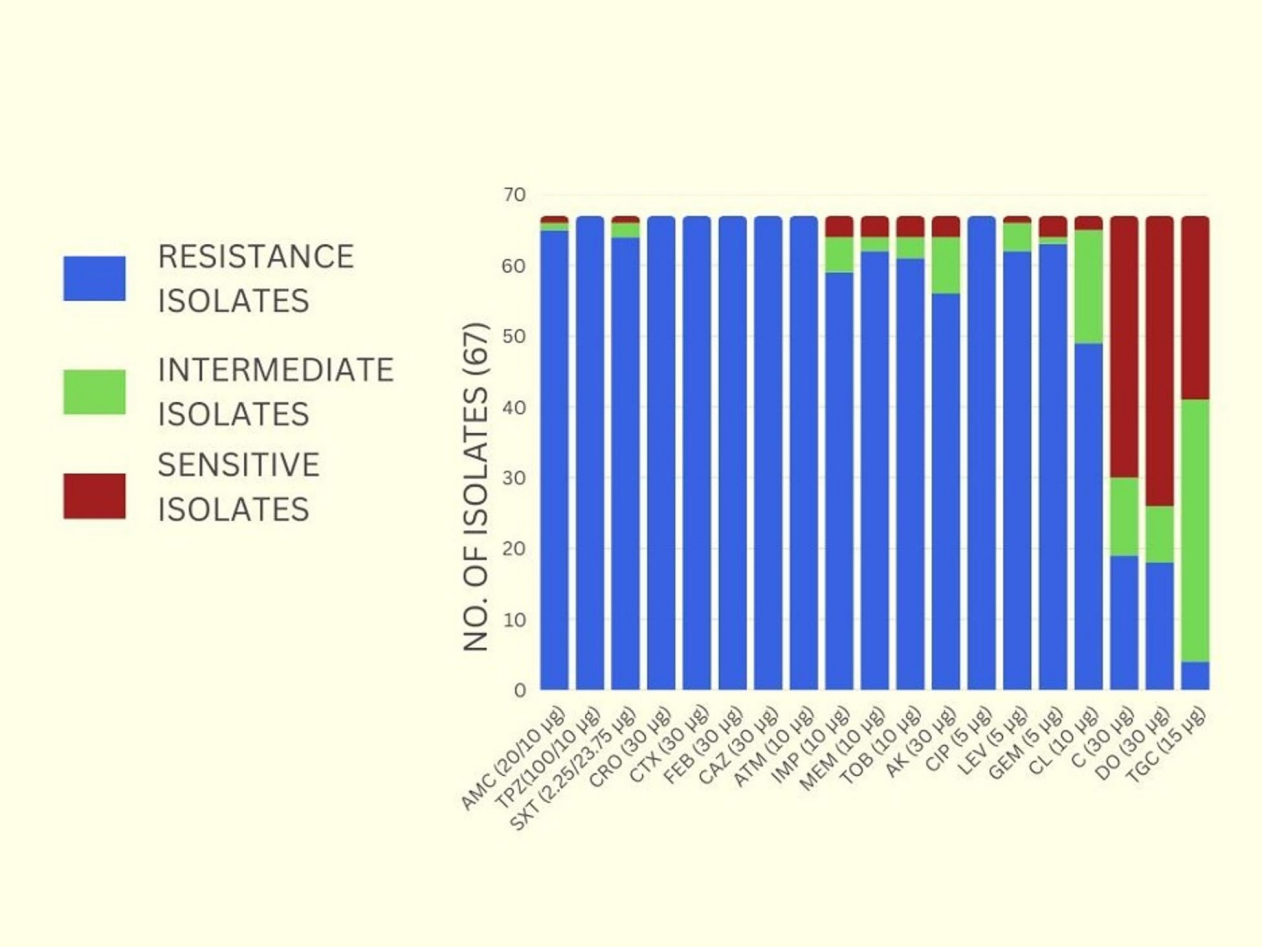
The antimicrobial susceptibility test of *Klebsiella pneumoniae* isolates against the tested antibiotics.

The antimicrobial susceptibility test revealed that most of the collected *K. pneumoniae* isolates exhibited an extensively drug-resistant (XDR) phenotype. They showed high resistance to several antibiotic groups, including β-lactams (e.g., amoxicillin-clavulanic acid, ceftriaxone, cefepime, and piperacillin-tazobactam), carbapenems (e.g., imipenem and meropenem), aminoglycosides (e.g., tobramycin and amikacin), and fluoroquinolones (e.g., ciprofloxacin and gemifloxacin). Moderate resistance was also observed to colistin, while low resistance was noted to chloramphenicol, doxycycline, and tigecycline, as shown in Table S2 and Fig. 1.

The results presented in Table S2 and Fig. 1 are supported by the isolate level data in Supplementary Table S3, which also provides the detailed resistance profile of each isolate together with the corresponding multiple antibiotic resistance (MAR) index. Results showed that 10 isolates (10/67; 14.9%) exhibited a MAR index from 0.58 to 0.74, while 57 (85.1%) isolates exhibited a MAR index from 0.79 to 1.00, as displayed in Table S3. As shown in Supplementary Table S4, four isolates (5.9%) were identified as multidrug-resistant (MDR), one isolate was identified as pan-drug resistant (PDR), and the remaining isolates (92.5%) were categorized as extensively drug-resistant (XDR).

### Genetic diversity using ERIC-PCR

**Fig. 2:**
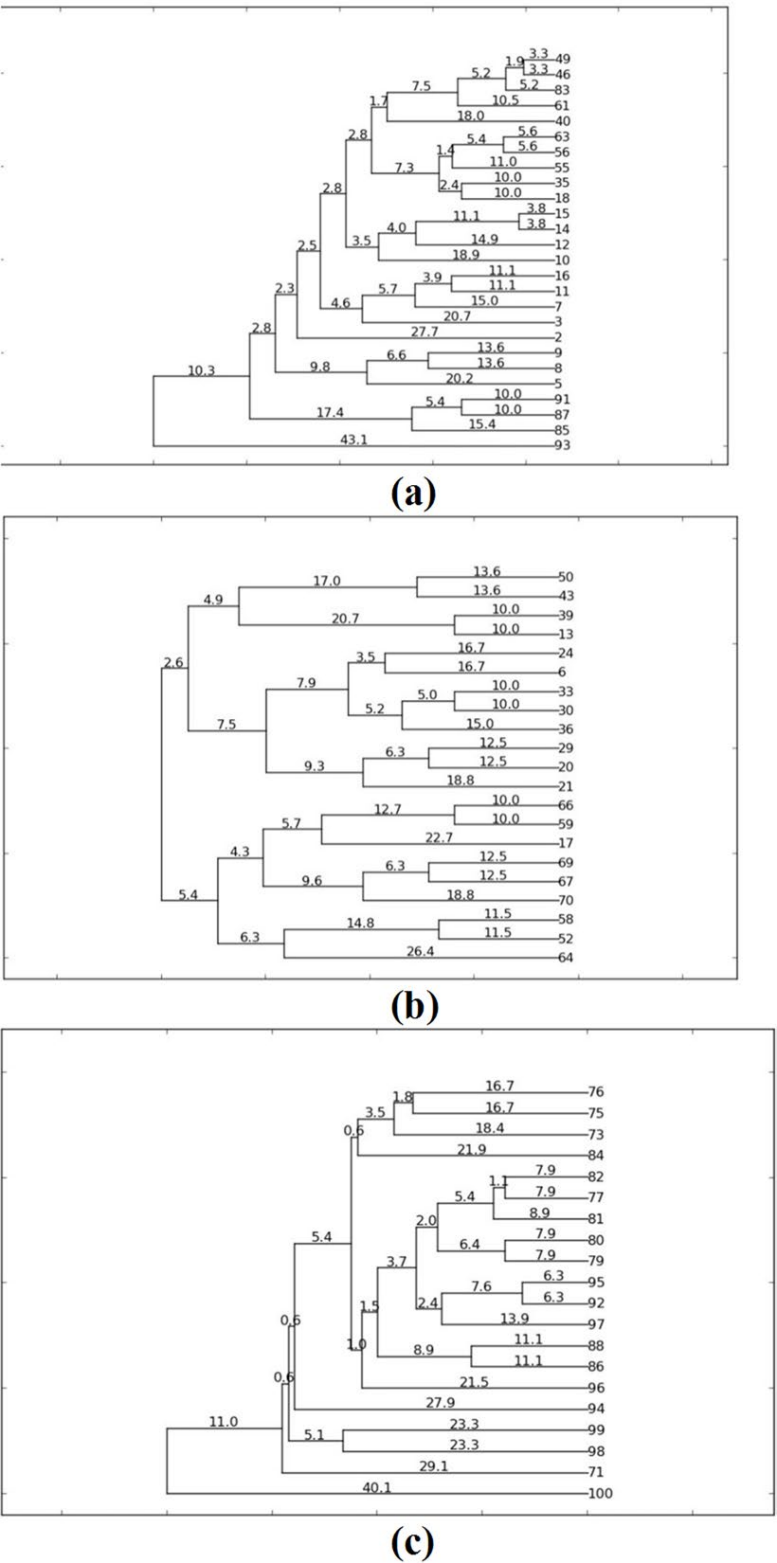
The dendrograms were constructed using PyElph 1.4 to demonstrate the levels of similarity among the collected *Klebsiella pneumoniae* clinical isolates. **(a).** Dendrogram 1; **(b)**. Dendogram 2; **(c)**: Dendogram 3.

For this study, a total of 67 *K. pneumoniae* isolates were analyzed for clonal relationship using ERIC-PCR, as shown in Figure S1. The dendrograms were constructed using PyElph 1.4 to demonstrate the levels of similarity among the isolates. As indicated in dendrograms (Fig. 2a–c), various clustering patterns of the isolates were observed. The analysis revealed the identification of different numbers of clusters, each with distinct patterns.

According to Table S5a, the similarity indices of 26 isolates analyzed by dendrogram 1 (Fig. 2a) were calculated. This dendrogram contained 25 merges and 50 branches and showed one large primary cluster composed of 25 closely related isolates, with similarity indices ranging from 93.4% to 69.2%, and one isolate (No. 93) forming a distinct outlier branch with a similarity index of 13.8%. The large primary cluster was further subdivided into approximately ten distinct sub-clusters based on their genetic similarity. Cluster 1 contained isolates 49, 46, and 83, which were very close (similarity = 93.4–89.6%). Cluster 2 included isolates 63, 56, and 55 (similarity = 88.8–77%). Cluster 3 comprised isolates 35 and 18 (similarity = 80%). Cluster 4 contained isolates 15, 14, 12, and 10 (similarity = 92.4–62.2%). Cluster 5 consisted of isolates 16, 11, 7, and 3 (similarity = 77.8–58.6%). Cluster 6 comprised isolates 9, 8, and 5 (similarity = 72.8–59.6%). Cluster 7 included isolates 91, 87, and 85 (similarity = 80–69.2%). Clusters 8, 9, and 10 represented single isolates (61, 40, and 2, respectively) with relatively low similarities to the rest of the dataset (79%, 64%, and 44.6%, respectively). Although several isolates such as 49, 55, 18, 15, 10, 7, 3, 9, and 91 exhibited relatively close genetic similarity to other members within their respective clusters, they were excluded from further analysis to avoid redundancy. Representative isolates from each cluster were instead selected based on their reproducible ERIC-PCR profiles and distinctive phenotypic resistance patterns, ensuring that each selected strain reflected the genetic and phenotypic diversity within its group. Overall, the dendrogram indicated a high level of genetic diversity among the 26 isolates, which were distributed into approximately ten clusters (93, 85, 83, 63, 61, 40, 16, 12, 5, and 2). This diversity reflects the wide range of similarity indices, representing distinct genetic patterns among the studied strains. According to Table S5b, the similarity indices of 20 isolates analyzed by dendrogram 2 (Fig. 2b) were calculated. This dendrogram contained 19 merges and 38 branches. The isolates were grouped into six large clusters based on their similarity indices. The largest cluster included isolates 82, 77, 81, 80, 79, 95, 92, 97, 88, 86, and 96 with relatively high similarity indices ranging from 84.2% to 57%. On the other hand, some formed smaller clusters such as (75, 76, 73, 84), whose similarity indices ranged between 66.6% and 56.2%, while others appeared as single branches such as isolate 94 (similarity = 44.2%), 98 and 99 (similarity = 53.4%), 71 (similarity = 41.8%), and significantly isolate 100, being the most genetically distant with a similarity index of 19.8%. Although isolate number 97 shared moderate genetic similarity (72.2%) with the (92, 95) cluster, it was selected as a representative isolate due to its reproducible ERIC-PCR profile and distinctive phenotypic resistance pattern, making it a suitable candidate for downstream molecular and antimicrobial characterization. Overall, the dendrogram indicated high genetic diversity among the isolates, forming approximately 13 clusters (100, 99, 97, 96, 94, 86, 84, 81, 80, 77, 75, 73, 71). This diversity reflects the wide range of similarity indices, representing distinct genetic patterns among the analyzed strains.

According to Table S5c, the similarity indices of 21 isolates analyzed by dendrogram 3 (Fig. 2c) were calculated. This dendrogram contained 20 merges and 40 branches. The isolates clustered into four major clusters: clusters no. (50, 43, 39, 13) with similarity indices ranging between 72.8% and 80%; clusters no. (24, 6, 33, 30, 36, 29, 20, 21) with similarity indices ranging between 62.4% and 80%; clusters no. (66, 59, 17, 69, 67, 70) with similarity indices ranging between 54.6% and 80%; and clusters no. (58, 52, 64) with similarity indices ranging between 47.2% and 77%. Although isolates No. 6, 29, and 21 in cluster 2, as well as isolates number 66, 17, 69, 67, and 70 in cluster 3, appeared closely related to isolates 36 and 59, respectively, since their similarity indices were relatively close, these isolates were excluded from further experimental analysis to avoid redundancy. Isolates number 36 and 59 were selected as representative strains for their respective genetic groups owing to their consistent ERIC-PCR banding profiles and unique phenotypic resistance characteristics, rendering them reliable representatives for subsequent molecular and antimicrobial investigations. The number of clusters and their similarities varied, reaching a total of six clusters containing 21 isolates that differed from one another, indicating a higher level of genetic diversity (64, 59, 58, 50, 36, and 39). Overall, the results indicate that there are both clonal relatedness and genetic diversity among the isolates that were tested. Therefore, there was evidence of a mixture of community strains and outbreak strains. The investigators selected 29 isolates with diverse ERIC-PCR profiles, high levels of genetic diversity, and multi-drug resistance for further characterization in order to provide appropriate representation of the population and clinically relevant isolates for future experimental studies.

### Detection of antibiotic-resistant genes

**Fig. 3:**
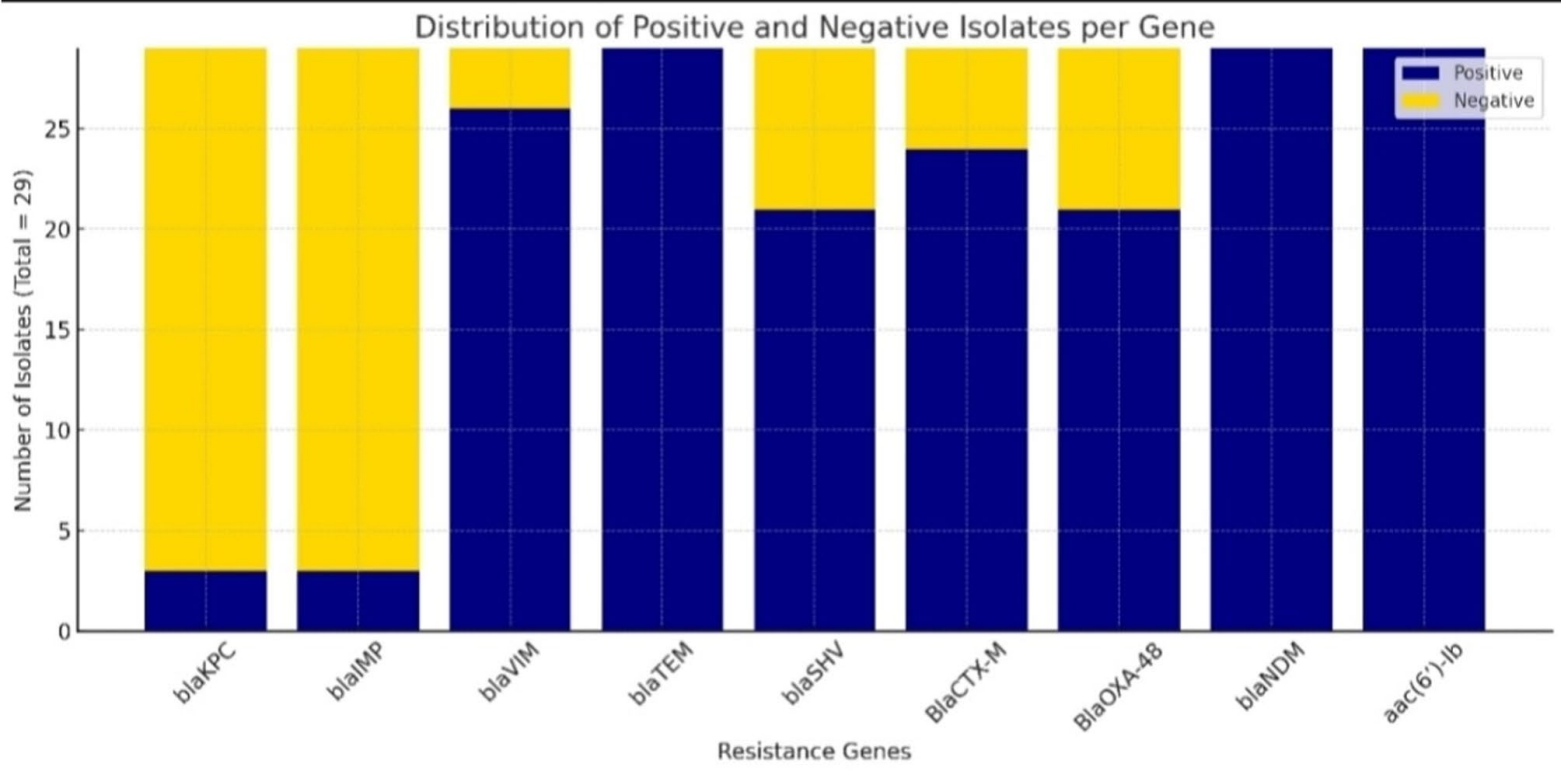
Presence or absence of the resistant genes in *Klebsiella pneumoniae* isolates.

The nine resistance genes *bla*SHV, *bla*KPC, *bla*IMP, *bla*VIM, *bla*TEM, *bla*NDM, *bla*CTX-M, *bla*OXA-48, and *aac*(6’)-Ib were detected in the 29 XDR *K. pneumoniae* isolates (Fig. 3). These findings underscore a high prevalence of resistance genes contributing to the antimicrobial resistance patterns observed in the isolates, which are mostly classified as extensively drug-resistant (XDR). The antibiotic-resistant genes that were detected in each of the 29 isolates are displayed in Table 1. The chi-square tests were performed to assess the association between the number of isolates and the presence of resistance genes (*bla*SHV, *bla*KPC, *blaI*MP, *bla*VIM, *bla*CTX-M, and *bla*OXA-48) in 29 *K. pneumoniae* isolates. All genes showed a Pearson chi-square value of 29.000 with 28 degrees of freedom (df) and a p-value of 0.424, indicating no significant association between the presence of these genes and the number of isolates. The genes *bla*TEM, *bla*NDM, and *aac*(6’)-Ib were present in all isolates.

**Table 1:** Antibiotic-resistant genes detected among extensively drug-resistant (XDR) *K. pneumoniae* isolates (*n* = 29).

Isolate Nr	Isolate code	Antibiotic-resistant genes								
		blaKPC	blaIMP	blaVIM	blaTEM	blaSHV	blaCTX-M	blaOXA-48	blaNDM	aac(6’)-Ib
1	K2	-	-	-	+	-	-	-	+	+
2	K5	-	-	+	+	-	-	-	+	+
3	K12	-	-	+	+	-	-	+	+	+
4	K16	-	-	+	+	-	-	-	+	+
5	K36	-	-	+	+	+	-	+	+	+
6	K39	-	-	+	+	+	+	-	+	+
7	K40	-	-	+	+	-	+	+	+	+
8	K50	+	-	+	+	+	+	+	+	+
9	K58	-	-	+	+	+	+	+	+	+
10	K59	-	-	+	+	+	+	+	+	+
11	K61	-	+	+	+	+	+	-	+	+
12	K63	-	-	+	+	+	+	-	+	+
13	K64	-	+	+	+	+	+	+	+	+
14	K71	-	-	+	+	+	+	+	+	+
15	K73	-	-	+	+	+	+	+	+	+
16	K75	-	-	+	+	+	+	+	+	+
17	K77	-	+	-	+	+	+	+	+	+
18	K80	-	-	+	+	+	+	+	+	+
19	K81	-	-	+	+	+	+	+	+	+
20	K83	-	-	+	+	+	-	+	+	+
21	K84	+	-	+	+	+	+	+	+	+
22	K85	-	-	+	+	+	+	-	+	+
23	K86	-	-	+	+	+	+	-	+	+
24	K93	-	-	+	+	+	+	+	+	+
25	K94	-	-	+	+	-	+	+	+	+
26	K96	-	-	-	+	-	+	+	+	+
27	K97	+	-	+	+	-	+	+	+	+
28	K99	-	-	+	+	+	+	+	+	+
29	K100	-	-	+	+	+	+	+	+	+

### Synthesis and characterization of silver nanoparticles (AgNPs)

**Fig. 4:**
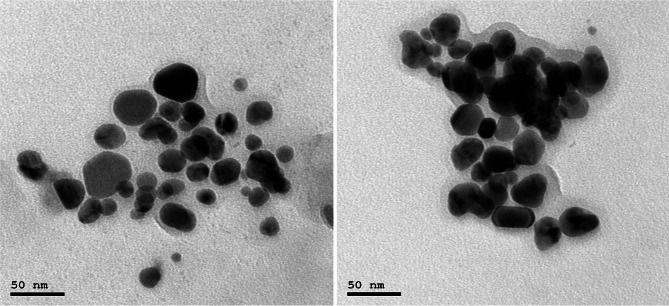
Transmission electron microscope analysis (TEM) of silver nanoparticles (AgNPs) with particle size distribution from approximately 5 nm to 40 nm, with an average particle size of 19.68 nm.

**Fig. 5:**
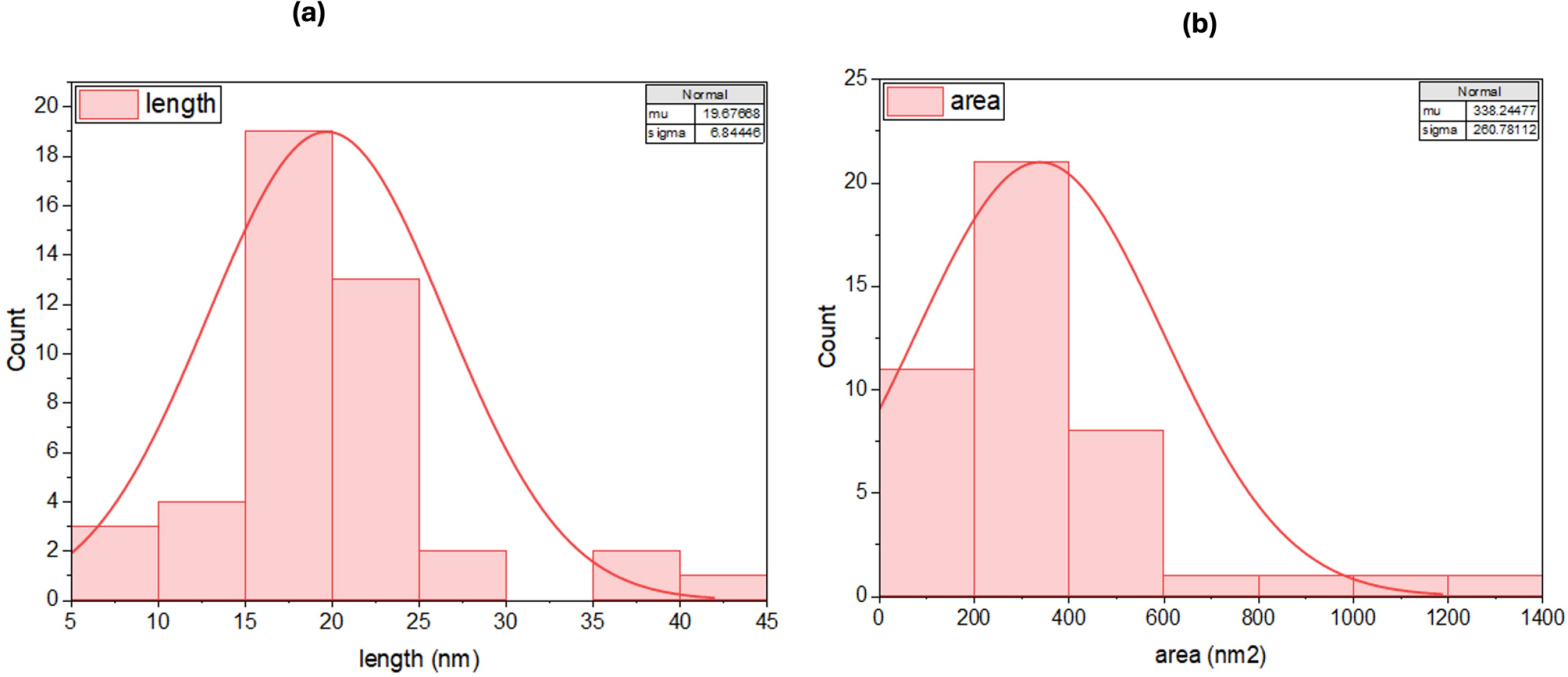
Histogram analysis of silver nanoparticles (AgNPs) showing (a) particle size distribution and (b) surface area, determined using Origin software.

**Fig. 6:**
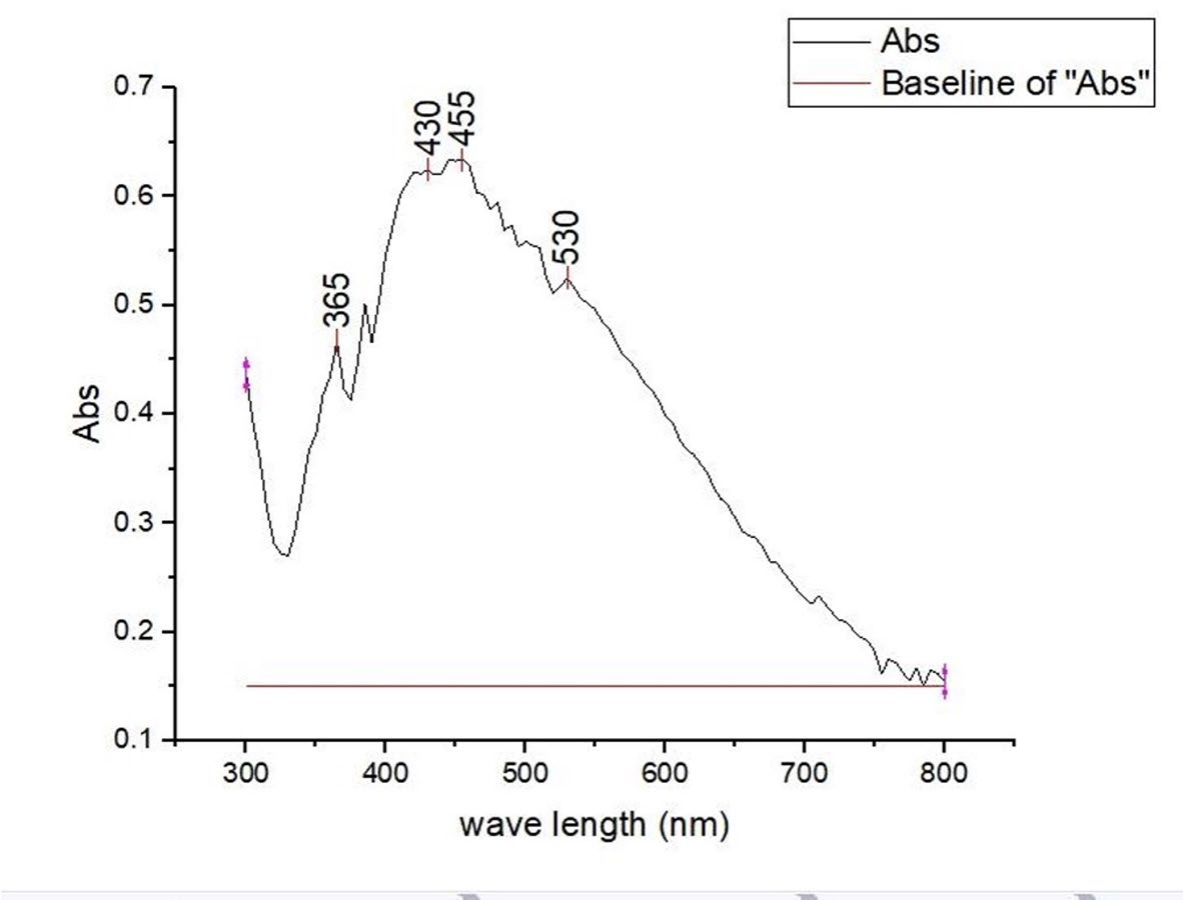
UV-Vis absorption spectrum analysis of silver nanoparticles (AgNPs) using the Origin software.

**Fig. 7:**
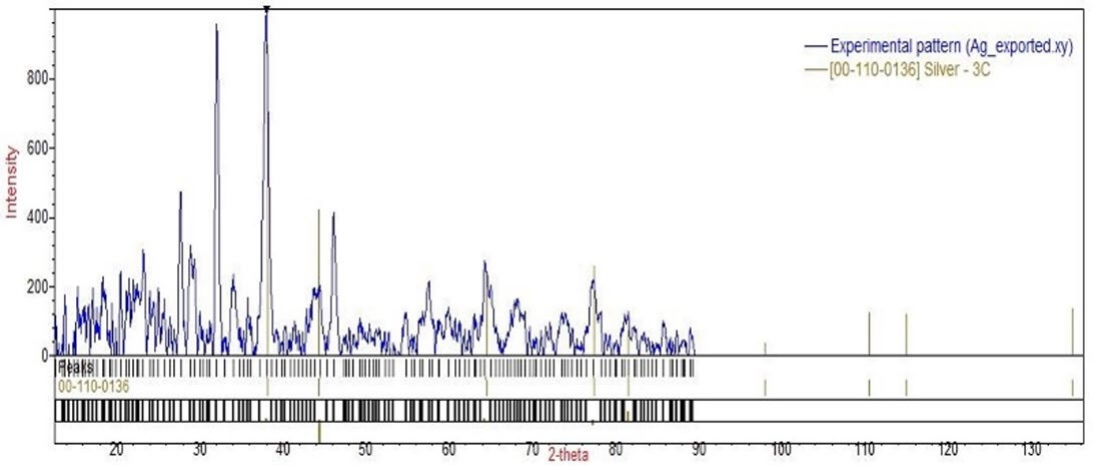
X-ray diffraction (XRD) analysis of silver nanoparticles (AgNPs) compared with COD using Qualx software.

The synthesis of AgNPs was verified by observing a color change from colorless to a dark brown solution within 5–10 min, indicating the presence of AgNPs. Characterization of the nanoparticles was achieved through TEM, UV spectrophotometer analyses, and XRD. The TEM images illustrated spherical particles as shown in Fig. 4. Moreover, subsequent analysis using ImageJ and Origin software revealed a particle size distribution from 5 to 40 nm, an average diameter of 19.68 ± 6.84 nm, and a surface area of 338 nm^2^, as shown in Fig. 5. From the particle size, the mass concentration was calculated to be 1.348 mg/mL (Table S6). The UV absorption spectrum showed the highest wavelength at 455 nm, representing the λmax and a wide peak, indicating that the AgNPs were of polydiverse nature (Fig. 6). Thus, the multiple absorption peaks observed in the UV–Vis spectrum are consistent with the heterogeneous particle distribution visualized in the TEM micrographs. The analysis of the XRD data in Fig. 7 revealed characteristic diffraction peaks at 2θ (38.005°, 44.489°, 64.291°, 77.26°, and 81.5°). These peaks were compared with the Crystallography Open Database (COD) file number (00-110-0136), which is associated with the face-centered cubic (fcc) forms of metallic silver, using the Qualx software. In addition, the average grain size of AgNPs was determined using Scherrer’s equation, and the results are shown in Table S7. The average grain size of AgNPs is 18.84 nm based on the XRD data analysis.

### Determination of antimicrobial activity of AgNPs

The results of the agar-well diffusion method showed the presence of inhibition zones, indicating the effectiveness of AgNPs against *K. pneumoniae* isolates. The size of the inhibition zones of AgNPs ranged from 14.5 nm to 23.5 mm across the 29 tested XDR isolates, with no inhibition zone of the control, as shown in Table S8. However, when comparing the inhibition zones (mm) of meropenem alone to those obtained upon its combination with AgNPs, results showed that all isolates (*n* = 29) exhibited a significant increase in the average inhibition zone (Table S8). For quantitative determination using the checkerboard assay, the MIC of meropenem alone was found to be 12.5 µg/mL for all tested isolates. The results for all the isolates are shown in Table S9. According to this table, the combination showed a partial synergistic effect of 34.5% (10/29) against isolates K5, K12, K71, K77, K80, K81, K85, K86, K96, and K97, while it showed a synergistic effect of 65.5% (19/29) against the remaining isolates.

## Discussion

This study aimed to analyze antimicrobial resistance patterns in clinical isolates as well as the effectiveness of AgNPs as antimicrobial agents when combined with meropenem against multidrug-resistant (MDR) *K. pneumoniae* pathogens. As the first step in this study, a total of one hundred Gram-negative bacteria were collected and identified by Gram stain, culture, and biochemical tests. All isolates were short Gram-negative rods, and 67 strains proved to be *K. pneumoniae*. For further confirmation, the identification was validated using the VITEK 2 automated identification system. All 67 isolates were consistently recognized as *K. pneumoniae* with high confidence levels between 96 and 99%, confirming both the accuracy and reproducibility of the identification techniques employed. The results of antimicrobial susceptibility testing, much like previous research, highlighted a concerning level of antibiotic resistance in *K. pneumoniae* isolates. Most of the isolates tested displayed a high percentage of resistance against antibiotics, especially cephalosporins and β-lactams. Interestingly, all isolates were resistant to agents such as cefotaxime, ceftazidime, and ceftriaxone, in agreement with findings by Taha et al., who reported more than 98% rates of resistance for these agents^40^. Carbapenems such as imipenem and meropenem, although previously considered last-line agents for treatment, also showed high resistance and low to moderate sensitivity in our isolates^40^. These findings are consistent with Hassuna et al.^41^ and Attia NM et al.^42^, who reported carbapenem resistance rates greater than 90% in some cases. However, our resistance rates for these drugs were comparatively lower, which could be due to geographical or temporal variations in antimicrobial usage and resistance development. Our isolates also showed high resistance and low to moderate sensitivity to amikacin, in agreement with Hassuna et al., who reported aminoglycoside resistance above 95%^41^. However, this contrasts with the previous investigators who observed the better effectiveness of amikacin against MDR *K. pneumoniae* isolates^12,43^. It also differs from El-Din et al., who described an intermediate response to aminoglycosides^44^. On the other hand, antibiotics such as colistin and tigecycline proved to be more effective, showing a significantly higher percentage of sensitive isolates, which is in line with previous studies^45,46^. Despite some recent reports indicating rising resistance to colistin and despite our findings of high resistance and only low to moderate sensitivity, colistin remains a viable last-line treatment option, especially for life-threatening MDR infections.

Overall, the antimicrobial susceptibility tests indicate that 92.5% of the studied bacterial isolates exhibit an extensive XDR phenotype, and one isolate exhibits a PDR phenotype to commonly used antibiotics, compared with the previous studies. According to Attia et al., out of 142 clinical isolates, 47 *K. pneumoniae* strains were studied. Among these, 47.8% were classified as XDR, 41.3% as MDR, and the rest remained susceptible to the most used antibiotics^37^. Our findings showed that 10 isolates (10/67; 14.9%) exhibited a MAR index from 0.58 to 0.74, while 57 (85.1%) isolates exhibited a MAR index from 0.79 to 1.00, indicating a high antibiotic resistance profile of the collected clinical isolates.

Similarly, Mohamed et al. analyzed 100 clinical *K. pneumoniae* isolates, including 36 classical strains (24% MDR, 4% XDR, 8% PDR) and 64 hypervirulent strains, which showed higher resistance rates: 36% MDR, 14% XDR, and 14% PDR^22^. These findings point to a worrying rise in antimicrobial resistance, especially among hypervirulent strains, highlighting the urgent need for stronger surveillance systems, responsible antibiotic use, and the development of new and effective treatment options. This study explored the genetic diversity and epidemiological links among *K. pneumoniae* isolates using the ERIC-PCR technique. A total of 67 isolates were examined to determine the genetic relationship between isolates. The findings of this study showed a high level of genetic variation, as the isolates were distributed across multiple distinct clusters formed in the three dendrograms.

Similarity indices varied widely, from a minimum of 13.8% to a maximum of 93.4%, and were indicative of the high genetic diversity of the collection. Based on their distinct clustering patterns and potential epidemiological relevance, 29 of these isolates were chosen for more detailed analysis. These findings agree with previous studies, which recognized ERIC-PCR as a valuable tool for discrimination among *K. pneumoniae* strains. For example, Parsaie Mehr et al. tested 35 clinical isolates with ERIC-PCR and reported 32 varied genetic profiles, of which 28 were non-clustered, which suggested a non-clonal and predominantly heterogeneous population^47^. Further, Moosavian and Emam tested 26 isolates and identified 23 ERIC types, of which there were 11 clusters and 20 singletons^46^. They established through their research that 76.9% of the isolates were genetically unique, with 23% sharing any genetic similarities. According to this study, it proved that the colistin-resistant strains included in their ERIC-PCR analysis were not genetically related, effectively ruling out the possibility of a clonal outbreak. Likewise, Attia et al. have reported high genetic diversity among *K. pneumoniae* isolates, clustering 46 isolates into three predominant clusters and identifying 30 various ERIC genotypes^42^. Their findings also suggest a polyclonal distribution pattern of *K. pneumoniae* within the hospital setting, reflecting the results that were observed in the present study.

Our study supports these earlier findings. The dendrograms we generated consistently revealed both clustered isolates, likely indicating possible nosocomial transmission, as seen in Osama et al.^43^, as well as genetically unique, sporadic strains. Notably, it stood out as highly distinct, hinting at the possible emergence of community-acquired strains. These results echo other studies, e.g., Ferreira et al., which emphasize *K. pneumoniae’s* remarkable genetic flexibility and its ability to evolve and adapt^48^. These results highlight the complexity of the *K. pneumoniae* population structure in the clinic and validate the use of ERIC-PCR as a molecular epidemiology tool. Tracking the spread of *K. pneumoniae* strains, spotting likely outbreaks, and putting into practice efficient infection control and prevention procedures all require an awareness of such patterns of genetic diversity.

To determine the mechanism of resistance, the presence of several resistance genes in our isolates was tested. Resistance genes identified were *bla*IMP, *bla*SHV, *bla*TEM, *bla*CTX-M, *bla*NDM, *bla*VIM, *bla*OXA-48, and *bla*KPC, each contributing to beta-lactam antibiotic resistance. Every isolate carried one or more of these resistant genes, indicating a high rate of MDR occurrence of *K. pneumoniae* isolates that may spread further to XDR strains. The high occurrence of these genes is significant because it means the presence of carbapenem-resistant genes (CREs) and ESBL genes in *Enterobacteriaceae*, with different percentages in each isolate. In our study, for CREs, *bla*NDM was found in all isolates, *bla*VIM was detected in 89% of isolates, *bla*IMP was the second least prevalent gene, and *bla*KPC was found to be the least prevalent gene among *K. pneumoniae* isolates. In comparison with other studies like Hassuna et al.^41^, Zafer et al.^49^, and Hussein et al.^50^, they have revealed higher expression levels of *bla*KPC among isolates. Taha et al. found that 15% of isolates carried *bla*VIM, 7.5% carried *bla*IMP, and 3.8% carried *bla*NDM^40^. Whereas Zafer et al. indicated a moderate presence of *bla*NDM and no presence of *bla*VIM^49^. On the other hand, blaOXA-48 was found to be the third least common gene among *K. pneumoniae* isolates in our study. This is in contrast with previous studies by Taha et al., who found blaOXA-48 as the most frequently occurring gene in Egypt^40^, and Raheel et al. demonstrated that the *bla*OXA-48 gene was the most frequently occurring, at approximately 96.2% in *K. pneumoniae* isolates^51^. For ESBL, *bla*TEM was found in all isolates, *bla*CTX-M was detected in 79% of isolates, and *bla*SHV was detected in 72% of all isolates. Compared with the previous studies, Maleki et al.^45^, Ferreira et al.^48^, and Hassuna et al.^41^ reported a high prevalence of ESBL genes. However, there is a variability in the diversity of the resistant genes *bla*SHV and *bla*TEM in these studies. In Maleki et al. and Hassuna et al. studies, *bla*CTX-M was the most frequently occurring gene among ESBL genes^41,45^, while in Ferreira et al., *bla*TEM was the most frequently occurring gene among ESBL genes^48^. The resistant genes in MDR isolates show the genetic basis of antibiotic resistance and the challenges in managing this pathogen.

Nanoparticles are considered an excellent alternative to antibiotics, as they can solve the problem of MDR among bacteria, e.g., *K. pneumoniae*^52^. Among various nanometals used, AgNPs have shown strong, broad-spectrum antibacterial activity against microorganisms^53^. In several studies, AgNPs have been investigated for their role in combating XDR *K. pneumoniae*, and the findings of this study further confirm their effectiveness.

There are many ways to form (AgNPs) through the biological synthesis using living organisms (for example, plants, bacteria, fungi, algae, and yeast). The biological synthesis of AgNPs is a ‘green’, environmentally friendly, and efficient alternative to the formation of AgNPs using conventional chemical synthesis methods. When using biological synthesis, the reduction of the ionic form of the metal (Silver) into the Nanoscale Metal (Ag) is done with the aid of natural molecules contained in these organisms, such as terpenoids, flavonoids, ketones, aldehydes, amides, and carboxylic acids. This allows for a more controlled method of synthesizing NPs with Ag ions without having to use a large quantity of harmful reagents. Green synthesis (Plant-mediated biological methods) has received much attention as a method to produce AgNPs because it is sustainable, easy, and cost-effective^54^. It does not produce hazardous by-products, and therefore it is much more reliable and environmentally friendly. In addition to reducing silver ions to AgNPs, the Plant Extract will stabilize the AgNPs, allowing for precise control of NP size, shape, and dispersal^20,55,56^.

The phyto constituents of Camellia sinensis serve as reducing, capping, and stabilizing agent simultaneously, eliminating the need for synthetic chemicals and reducing the environmental impact associated with nanoparticles. This ensures the biocompatibility of AgNPs more sustainably and aligns with the modern trend of ecological responsibility in biomedical and nanotechnological applications worldwide^20^.

In this study, AgNPs were synthesized via green synthesis using *Camellia sinensis* (green tea) extract. The formation of AgNPs was visually confirmed through a distinctive color change from colorless to dark brown. Previous studies employed other plants for the synthesis of AgNPs, such as Dash et al.^57^, who used bay leaves, and Mekky et al.^58^, who used *Spinacia oleracea* for the AgNPs synthesis. The presence of a brownish color of the solution and surface plasmon resonance (SPR) in this study likewise indicated the successful formation of AgNPs.

For the characterization of AgNPs, a UV spectrophotometer was utilized for the determination of the optical properties of AgNPs and for determining the surface plasmon resonance (SPR). In this study, the UV-Vis spectrophotometry analysis showed an SPR peak at 455 nm, revealing the creation of polydisperse AgNPs, which is in accordance with^59^ because SPR is between 350 and 500 nm. It also agrees with other reports, e.g., Ali et al.^35^, who reported a broader SPR range (450–500 nm), and Dash et al.^57^, who reported SPR peaks in the range of 460–470 nm. However, some studies reported a somewhat lower SPR peak at 436 nm, such as Elsaid et al., who reported a peak at 420 nm^60^. These variations in SPR are owing to the difference in the phytochemical content of the plant extract, synthesis conditions, and nanoparticle size distribution. The crystallinity of the developed AgNPs was confirmed by XRD analysis, as evident peaks at 2θ = 38.005°, 44.489°, 64.291°, 77.26°, and 81.5° are attributed to the (111), (200), (220), and (311) crystallographic planes, respectively. These peaks are consistent with the standard data from the Crystallography Open Database (COD), file number 00-110-0136, which confirms the face-centered cubic (fcc) structure of metallic silver. These findings agree with previously published studies, as the same diffraction peaks for the same lattice planes were identified using the JCPDS card no. 04-0783. Other studies also observed the same diffraction patterns, validating that FCC is a general characteristic of biologically synthesized AgNPs^35,61^. Also, the grain size of AgNPs was calculated using the Scherrer equation, which is 18.92 nm, which is larger than the grain size of Nishibuchi et al.^62^, which is 3.42 nm, and larger than the grain size of Mogole et al.^61^ which ranges between 4.9 and 5.3 nm; these differences could be attributed to various concentrations of reducing agent, reaction time, and other experimental parameters.

Transmission Electron Microscopy (TEM) imaging in this study confirmed that the AgNPs were primarily spherical in shape with a particle size ranging from 5 to 55 nm and an average diameter of 19.68 nm. Comparatively, Nishibuchi et al.^62^ reported much smaller particles (~ 4.06 nm), while Ali et al.^35^ reported bigger particles (52 nm), and Elsaid et al.^60^ reported the size of the particle ranging from 5.39 to 23.8 nm, again indicating the influence of synthesis parameters and phyto-constituents of the plant. Generally, our findings are consistent with the broader literature on AgNPs. As spectral, morphological, and crystalline properties fall within expected values, differences in SPR peak position, particle size, and crystallite size among studies are indicative of the prevalent influence of synthesis protocols and plant extract composition. The use of *Camellia sinensis* in our study proved effective for nanoparticle synthesis with proper properties, which made it worthy of being a potential, green reducing, and stabilizing agent for nanoparticle synthesis.

In this study, the effect of AgNPs on MDR *K. pneumoniae* isolates was evaluated using qualitative and quantitative screening. Qualitative screening was carried out by the agar well diffusion technique, and results showed a maximum inhibition zone from 14.67 mm to 20 mm, which proves good antimicrobial activity. According to Mariselvam et al., inhibition zones were classified as follows: a diameter of < 9 mm was inactive, 9–12 mm partially active, and 13–18 mm active, acting as a control for the evaluation of the effectiveness of synthesized nanoparticles^63^. Logeswari et al. reported a 24 mm inhibition zone^64^, Jemal et al. observed zones of 13 mm and 14 mm^65^, and Mekky et al. recorded an impressive 34 mm, the highest among the studies^58^. These consistent results reinforce the idea that AgNPs are a strong candidate in the fight against MDR pathogens.

In the quantitative screening of our study, the MIC of AgNPs ranged from 336.18 µg/mL to 672.35 µg/mL, which was considered a relatively high MIC value. For *K. pneumoniae*, there was a synergistic and partially synergistic effect by applying AgNPs in combination with meropenem, showing FICI values of 0.375, 0.25, 0.5, and 0.75. Compared with the previous studies, Lopez-Carrizales et al. reported the MIC of AgNPs against *K. pneumoniae* at 4 µg/mL, and when AgNPs were combined with ampicillin and amikacin, the FICI values were recorded as 0.53 and 0.75, indicating partial synergy^39^. Dash et al. reported the MIC to be 12.5 µg/mL^57^. Desouky et al. established the MIC of AgNPs against *K. pneumoniae* as 8 µg/mL; when combined with ceftazidime, the FICI value was 0.23, indicating synergy, and when combined with amikacin, the FICI value was 0.75, indicating partial synergy^66^. Mekky et al. obtained an MIC of 4 µg/mL^58^. Khalil et al. also reported the MIC as 16 µg/mL, and when AgNPs were combined with piperacillin/tazobactam, FICI was 0.625, indicating partial synergy^38^. Ali et al. established the MIC of AgNPs against *K. pneumoniae* as 62.5 µg/mL^35^. Haji et al. reported the MIC as 64 µg/mL; with AgNPs in combination with ceftazidime, imipenem, ceftriaxone, and cefepime, FICI values were 0.25, 0.31, 0.13, and 0.5, respectively, and they indicated synergistic and partially synergistic effects^67^. Elsaid et al. reported the MIC ranged from 15.625 to 125 µg/mL^60^.

Further research is strongly recommended to validate the therapeutic potential and safety profile of AgNPs in vivo. This validation should begin with preliminary investigations using commercially available AgNPs in animal models and/or clinical trials to demonstrate that potential health benefits can be achieved under real-world conditions, while simultaneously confirming their safety for patient application. Continuous monitoring and more research are needed to address the persistent problem of bacteria becoming resistant to treatment and to identify the optimal use of AgNPs, including the correct dosage, size, and combination with other therapies to ensure maximum safety and efficacy against infectious agents.

To overcome the limitations of current studies, a concerted effort must be made to expand the fundamental experimental framework. This approach should include the incorporation of a wider variety of sample types and the performance of rigorous dose–response analyses. Crucially, future research should focus on understanding the detailed mechanisms Of action of AgNPs against resistant pathogens like *K. pneumoniae*. This involves elucidating the molecular pathways underlying their antimicrobial activity, particularly through molecular studies that explore their interaction with bacterial cell membranes. This integrated approach will provide deeper insight into the AgNP antimicrobial system and open new opportunities for clinical translation. Beyond fundamental and clinical validation, investigating the synergistic potential of AgNPs combined with conventional antibiotics, such as meropenem and others, is essential to determine the most effective combinations for clinical applications. Optimizing the appropriate dosage and concentration of AgNPs, whether used alone or in combination therapy, is critical to maximize antimicrobial efficacy while ensuring patient safety. Finally, establishing clear protocols for the clinical implementation of AgNPs will be key to facilitating their safe introduction into hospitals and minimizing risks to patients and healthcare staff. Moreover, long-term investigations are required to evaluate the environmental consequences of AgNPs and to generate evidence-based clinical guidelines that ensure their continued safe and sustainable application and responsible integration into medical practice.

## Conclusion

In this study, 67 *K. pneumoniae* clinical isolates were recovered from clinical settings in Egypt, of which 62 isolates (92.5%) exhibited XDR phenotypes. Antimicrobial susceptibility testing and detection of the major antibiotic-resistant genes, including CR and ESBL genes, followed by ERIC-PCR to confirm genetic relatedness, were carried out. *Camellia sinensis*-synthesized AgNPs exhibited strong antimicrobial activity against the collected 29 non-clonal XDR strains. The FICI analysis of 29 non-clonal XDR *K. pneumoniae* isolates proved enhanced antimicrobial activity with partial (34.5%) and complete synergistic (65.5%) effects in different isolates when AgNPs were combined with meropenem. Therefore, the combination of AgNPs with meropenem could be a useful alternative approach in combating XDR *K. pneumoniae*. Further studies are recommended for the use of this approach in clinical settings based on its benefits to enhance treatment outcomes. In vivo studies using animal models or clinical trials are strongly recommended to confirm the therapeutic potential and safety profile of AgNPs in real-world settings. Moreover, long-term studies should also be conducted to assess the environmental impact of AgNPs and develop detailed clinical guidelines to ensure their safe and sustainable use in healthcare environments.

## Supplementary Information

Below is the link to the electronic supplementary material.

Supplementary Material 1

## Data Availability

All data generated or analyzed during this study are included in this published article and supplementary file.
