# Supplementary material for: Camellia sinensis-synthesized silver nanoparticles and meropenem combination against extensively drug-resistant Klebsiella pneumoniae
Source: Sci Rep. 2026 Feb 20;16:7475. doi: 10.1038/s41598-026-38375-0 (PMC12929614; doi:10.1038/s41598-026-38375-0)
Supplement: Supplementary file 1 — Supplementary Material 1 [file 41598_2026_38375_MOESM1_ESM.docx]

**Table S1.** The primer sequences used in this study and the expected PCR product sizes.

| **Target gene** | **Primer** | **Primer sequence (5’-3’)** | **Annealing Temperature, °C (Ta)** | **PCR product size (bp)** | **Reference** |
| --- | --- | --- | --- | --- | --- |
| *blaKPC* | PF | ATGTCACTGTATCGCCGTCT | 55˚C | 829 | [24] |
|  | PR | TTTTCAGAGCCTTACTGCCC |  |  |  |
| *blaIMP* | PF | CATGGTTTGGTGGTTCTTGT | 53˚C | 488 |  |
|  | PR | ATAATTTGGCGGACTTTGGC |  |  |  |
| *blaVIM* | PF | AGTGGTGAGTATCCGACA | 53˚C | 280 |  |
|  | PR | ATGAAAGTGCGTGGAGAC |  |  |  |
| *blaTEM* | PF | ATCAGCAATAAACCAGC | 54˚C | 516 | [25] |
|  | PR | CCCCGAAGAACGTTTTC |  |  |  |
| *blaSHV* | PF | AGGATTGACTGCCTTTTTG | 54˚C | 392 |  |
|  | PR | ATTTGCTGATTTCGCTCG |  |  |  |
| *blaCTX-M* | PF | ATGTGCAGYACCAGTAARGTKATGGC | 54˚C | 593 | [26] |
|  | PR | TGGGTRAARTARGTSACCAGAAYCAGCGG |  |  |  |
| *blaOXA-48* | PF | TTGGTGGCATCGATTATCGG | 56˚C | 743 | [27] |
|  | PR | GAGCACTTCTTTTGTGATGGC |  |  |  |
| *bla NDM* | PF | AAC GGTTTGGCG ATCTGGTTT TC | 62.5˚C | 627 | [28] |
|  | PR | GGCGGAATG GCT CAT CAC GAT C |  |  |  |
| *aac(6’)-Ib* | PF | TTG CGATGCTCTATGAGTGGCTA | 55˚C | 482 | [29] |
|  | PR | CTCGAATGCCTGGCGTGTTT |  |  |  |

*bla*KPC, *bla*NDM, *bla*VIM, *bla*OXA-48 and *bla*IMP: gene coding for KPC, NDM, VIM, OXA-48-like, and IMP carbapenemases, respectively. *bla*CTX-m: gene coding for cefotaxime (CTX-M) extended-spectrum β-lactamase, *bla*TEM: gene coding for TEM extended-spectrum βlactamase, *bla*SHV: gene coding for SHV extended-spectrum β-lactamase, PF : forward primer, PR : reverse primer, Ta: annealing temperature.

**Table S2.** Antimicrobial resistance percentage pattern among clinical isolates (N=67)

| **Antimicrobial agents** | **Resistance pattern of clinical isolates**  **(N = 67)** | | |
| --- | --- | --- | --- |
|  | **Sensitive (S) Percentage** | **Intermediate (I)**  **Percentage** | **Resistant (R) Percentage** |
| Amoxicillin/Clavulanate (AMC) | 1.49% | 1.49% | 97.02% |
| Piperacillin/tazobactam (TPZ) | 0% | 0% | 100% |
| Trimethoprim/Sulfamethoxazole (SXT) | 1.49% | 2.98% | 95.53% |
| Ceftriaxone (CRO) | 0% | 0% | 100% |
| Cefotaxime (CTX) | 0% | 0% | 100% |
| Cefepime (FEB) | 0% | 0% | 100% |
| Ceftazidime (CAZ) | 0% | 0% | 100% |
| Aztreonam (ATM) | 0% | 0% | 100% |
| Imipenem (IMP) | 4.48% | 7.46% | 88.06% |
| Meropenem (MEM) | 4.48% | 2.98% | 92.54% |
| Tobramycin (TOP) | 4.48% | 4.48% | 91.04% |
| Amikacin (AK) | 4.48% | 11.94% | 83.58% |
| Ciprofloxacin (CIP) | 0% | 0% | 100% |
| Levofloxacin (LEV) | 1.49% | 5.97% | 92.54% |
| Gemifloxacin (GEM) | 4.48% | 1.49% | 94.03% |
| Colistin (CL) | 2.98% | 23.88% | 73.13% |
| Chloramphenicol (C) | 55.22% | 16.42% | 28.36% |
| Doxycycline (DO) | 61.19% | 11.94% | 26.87% |
| Tigecycline (TGC) | 38.81% | 55.22% | 5.97% |

**Table S3**. Antimicrobial susceptibility profile and multiple antibiotic resistance (MAR) index of the MDR *Klebsiella pneumoniae* clinical isolates

| Isolate no. | **MAR index** | **AMC** | **TPZ** | **CAZ** | **CRO** | **CTX** | **FEB** | **ATM** | **IMP** | **MEM** | **TOB** | **AK** | **CIP** | **LEV** | **GEM** | **SXT** | **CL** | **C** | **DO** | **TGC** |
| --- | --- | --- | --- | --- | --- | --- | --- | --- | --- | --- | --- | --- | --- | --- | --- | --- | --- | --- | --- | --- |
| KP2 | 0.79 | R | R | R | R | R | R | R | R | R | R | R | R | I | R | R | R | S | S | I |
| KP3 | 0.58 | R | R | R | R | R | R | R | R | R | I | I | R | S | S | R | I | S | S | S |
| KP5 | 0.84 | R | R | R | R | R | R | R | R | R | R | R | R | R | R | R | R | S | S | S |
| KP6 | 0.84 | R | R | R | R | R | R | R | R | R | R | R | R | R | R | R | R | S | S | S |
| KP7 | 0.95 | R | R | R | R | R | R | R | R | R | R | R | R | R | R | R | R | R | R | I |
| KP8 | 0.84 | R | R | R | R | R | R | R | R | R | R | R | R | R | R | R | I | R | I | S |
| KP9 | 0.89 | R | R | R | R | R | R | R | R | R | R | R | R | R | R | R | I | R | R | I |
| KP10 | 0.89 | R | R | R | R | R | R | R | R | R | R | R | R | R | R | R | I | I | R | R |
| KP11 | 0.74 | R | R | R | R | R | R | R | R | R | S | S | R | R | R | R | R | I | S | I |
| KP12 | 0.74 | R | R | R | R | R | R | R | R | R | I | I | R | R | R | R | R | I | S | I |
| KP13 | 0.84 | R | R | R | R | R | R | R | R | R | R | R | R | R | R | R | R | S | I | I |
| KP14 | 0.63 | R | R | R | R | R | R | R | I | R | S | I | R | R | R | R | I | I | S | S |
| KP15 | 0.89 | R | R | R | R | R | R | R | R | R | R | S | R | R | R | R | R | R | R | I |
| KP16 | 0.84 | R | R | R | R | R | R | R | R | R | R | R | R | R | R | R | I | I | S | I |
| KP17 | 0.74 | R | R | R | R | R | R | R | I | R | R | R | R | R | R | I | I | S | S | S |
| KP18 | 0.84 | R | R | R | R | R | R | R | R | R | R | R | R | R | R | R | R | S | S | I |
| KP20 | 0.84 | R | R | R | R | R | R | R | R | R | R | R | R | R | R | R | R | S | S | I |
| KP21 | 1 | R | R | R | R | R | R | R | R | R | R | R | R | R | R | R | R | R | R | R |
| KP24 | 0.79 | R | R | R | R | R | R | R | R | R | R | R | R | R | R | I | R | S | S | I |
| KP29 | 0.74 | R | R | R | R | R | R | R | S | S | R | R | R | R | R | R | R | S | S | S |
| KP30 | 0.89 | R | R | R | R | R | R | R | R | R | R | R | R | R | R | R | R | R | I | I |
| KP33 | 0.84 | R | R | R | R | R | R | R | R | R | R | R | R | R | R | R | R | S | S | I |
| KP35 | 0.84 | R | R | R | R | R | R | R | R | R | R | R | R | R | R | R | R | S | I | I |
| KP36 | 0.95 | R | R | R | R | R | R | R | R | R | R | R | R | R | R | R | R | R | R | I |
| KP39 | 0.89 | R | R | R | R | R | R | R | R | R | R | R | R | R | R | R | I | S | R | S |
| KP40 | 0.79 | R | R | R | R | R | R | R | R | R | R | R | R | R | R | R | I | S | S | S |
| KP43 | 0.74 | R | R | R | R | R | R | R | R | R | R | R | R | I | I | R | R | S | S | S |
| KP 46 | 0.84 | R | R | R | R | R | R | R | R | R | R | R | R | R | R | R | R | S | S | S |
| KP49 | 0.89 | R | R | R | R | R | R | R | R | R | R | R | R | R | R | R | R | R | S | S |
| KP50 | 0.84 | R | R | R | R | R | R | R | R | R | R | R | R | R | R | R | R | I | S | I |
| KP52 | 0.84 | R | R | R | R | R | R | R | R | R | R | R | R | R | R | R | R | S | S | S |
| KP55 | 0.89 | R | R | R | R | R | R | R | R | R | R | R | R | R | R | R | R | R | S | S |
| KP56 | 0.84 | R | R | R | R | R | R | R | R | R | R | R | R | R | R | R | R | S | S | S |
| KP58 | 0.84 | R | R | R | R | R | R | R | R | R | R | R | R | R | R | R | R | S | S | S |
| KP59 | 0.84 | R | R | R | R | R | R | R | R | R | R | I | R | R | R | R | I | R | S | S |
| KP61 | 0.84 | R | R | R | R | R | R | R | R | R | R | R | R | R | R | R | R | S | S | I |
| KP63 | 0.84 | R | R | R | R | R | R | R | R | R | R | R | R | R | R | R | R | S | S | I |
| KP64 | 0.89 | R | R | R | R | R | R | R | S | S | R | I | R | R | R | R | R | R | R | R |
| KP66 | 0.84 | R | R | R | R | R | R | R | R | R | R | R | R | R | R | R | R | S | S | I |
| KP67 | 0.84 | R | R | R | R | R | R | R | R | R | R | R | R | R | R | S | R | S | S | I |
| KP69 | 0.95 | R | R | R | R | R | R | R | R | R | R | R | R | R | R | R | R | S | R | R |
| KP70 | 0.95 | R | R | R | R | R | R | R | R | R | R | R | R | R | R | R | R | R | R | I |
| KP71 | 0.74 | R | R | R | R | R | R | R | R | R | S | S | R | R | R | R | R | S | R | I |
| KP73 | 0.84 | R | R | R | R | R | R | R | R | R | R | R | R | R | R | R | R | S | S | I |
| KP75 | 0.79 | R | R | R | R | R | R | R | R | R | R | R | R | R | R | R | I | I | S | S |
| KP76 | 0.79 | R | R | R | R | R | R | R | R | R | R | I | R | R | R | R | I | S | S | S |
| KP77 | 0.79 | R | R | R | R | R | R | R | I | R | R | R | R | R | R | R | I | S | S | S |
| KP79 | 0.89 | R | R | R | R | R | R | R | R | R | R | R | R | R | R | R | R | R | I | I |
| KP80 | 0.68 | I | R | R | R | R | R | R | R | I | R | R | R | R | S | R | R | S | R | I |
| KP81 | 0.58 | R | R | R | R | R | R | R | I | S | R | I | R | I | S | R | R | I | I | I |
| KP82 | 0.95 | R | R | R | R | R | R | R | R | R | R | R | R | R | R | R | R | R | R | I |
| KP83 | 0.89 | R | R | R | R | R | R | R | R | R | R | R | R | R | R | R | R | S | R | I |
| KP84 | 0.84 | R | R | R | R | R | R | R | R | R | R | R | R | R | R | R | R | I | S | I |
| KP85 | 0.79 | R | R | R | R | R | R | R | I | R | R | R | R | R | R | R | R | S | S | S |
| KP86 | 0.84 | R | R | R | R | R | R | R | R | R | R | R | R | R | R | R | R | S | S | I |
| KP87 | 0.95 | R | R | R | R | R | R | R | R | R | R | R | R | R | R | R | R | R | R | I |
| KP88 | 0.79 | R | R | R | R | R | R | R | R | R | R | R | R | R | R | R | I | S | S | S |
| KP91 | 0.89 | R | R | R | R | R | R | R | R | R | R | R | R | R | R | R | R | R | I | I |
| KP92 | 0.84 | R | R | R | R | R | R | R | R | R | R | R | R | R | R | R | R | S | I | I |
| KP93 | 0.58 | S | R | R | R | R | R | R | S | I | I | I | R | I | R | R | R | S | S | I |
| KP94 | 0.89 | R | R | R | R | R | R | R | R | R | R | R | R | R | R | R | S | R | R | S |
| KP95 | 0.79 | R | R | R | R | R | R | R | R | R | R | R | R | R | R | R | I | I | S | S |
| KP96 | 0.89 | R | R | R | R | R | R | R | R | R | R | R | R | R | R | R | S | R | R | S |
| KP97 | 0.84 | R | R | R | R | R | R | R | R | R | R | R | R | R | R | R | R | S | S | I |
| KP98 | 0.95 | R | R | R | R | R | R | R | R | R | R | R | R | R | R | R | R | R | R | S |
| KP99 | 0.79 | R | R | R | R | R | R | R | R | R | R | R | R | R | R | R | I | I | S | I |
| KP100 | 0.84 | R | R | R | R | R | R | R | R | R | R | R | R | R | R | R | R | S | S | I |

Amoxicillin/clavulanate (AMC), Piperacillin/tazobactam (TPZ), Ceftazidime (CAZ), Ceftriaxone (CRO), Cefotaxime (CTX), Cefepime (FEB), Aztreonam (ATM), Imipenem (IMP), Meropenem (MEM), Tobramycin (TOP), Amikacin (AK), Ciprofloxacin (CIP), Levofloxacin (LEV) , Gemifloxacin (GEM), Trimethoprim/Sulfamethoxazole (SXT), Colistin (CL), Chloramphenicol (C), Doxycycline (DO) and Tigecycline (TGC).

**Table S4.** Antimicrobial susceptibility profile for the 67 isolates of *Klebsiella pneumoniae*

| **Serial no.** | **Isolate code** | **Number of antibiotics** | | |
| --- | --- | --- | --- | --- |
|  |  | **Resistant** | **Intermediate Sensitivity** | **Sensitive** |
| 1 | KP2 | 15 | 2 | 2 |
| 2 | KP3 | 11 | 3 | 5 |
| 3 | KP5 | 16 | 0 | 3 |
| 4 | KP6 | 16 | 0 | 3 |
| 5 | KP7 | 18 | 1 | 0 |
| 6 | KP8 | 16 | 2 | 1 |
| 7 | KP9 | 17 | 2 | 0 |
| 8 | KP10 | 17 | 2 | 0 |
| 9 | KP11 | 14 | 2 | 3 |
| 10 | KP12 | 14 | 4 | 1 |
| 11 | KP13 | 16 | 2 | 1 |
| 12 | KP14 | 12 | 4 | 3 |
| 13 | KP15 | 17 | 1 | 1 |
| 14 | KP16 | 15 | 3 | 1 |
| 15 | KP17 | 13 | 3 | 3 |
| 16 | KP18 | 16 | 1 | 2 |
| 17 | KP20 | 16 | 1 | 2 |
| 18 | KP21 | 19 | 0 | 0 |
| 19 | KP24 | 15 | 2 | 2 |
| 20 | KP29 | 14 | 0 | 5 |
| 21 | KP30 | 17 | 2 | 0 |
| 22 | KP33 | 16 | 1 | 2 |
| 23 | KP35 | 16 | 1 | 2 |
| 24 | KP36 | 18 | 1 | 0 |
| 25 | KP39 | 16 | 1 | 2 |
| 26 | KP40 | 15 | 1 | 3 |
| 27 | KP43 | 14 | 2 | 3 |
| 28 | KP 46 | 16 | 0 | 3 |
| 29 | KP49 | 17 | 0 | 2 |
| 30 | KP50 | 16 | 1 | 2 |
| 31 | KP52 | 16 | 0 | 3 |
| 32 | KP55 | 18 | 0 | 1 |
| 33 | KP56 | 16 | 0 | 3 |
| 34 | KP58 | 16 | 0 | 3 |
| 35 | KP59 | 15 | 2 | 2 |
| 36 | KP61 | 16 | 1 | 2 |
| 37 | KP63 | 16 | 1 | 2 |
| 38 | KP64 | 16 | 1 | 2 |
| 39 | KP66 | 16 | 1 | 2 |
| 40 | KP67 | 15 | 1 | 3 |
| 41 | KP69 | 18 | 0 | 1 |
| 42 | KP70 | 18 | 1 | 0 |
| 43 | KP71 | 15 | 1 | 3 |
| 44 | KP73 | 16 | 1 | 2 |
| 45 | KP75 | 15 | 2 | 2 |
| 46 | KP76 | 14 | 2 | 3 |
| 47 | KP77 | 14 | 2 | 3 |
| 48 | KP79 | 17 | 2 | 0 |
| 49 | KP80 | 14 | 3 | 2 |
| 50 | KP81 | 11 | 6 | 2 |
| 51 | KP82 | 18 | 1 | 0 |
| 52 | KP83 | 17 | 1 | 1 |
| 53 | KP84 | 16 | 2 | 1 |
| 54 | KP85 | 15 | 1 | 3 |
| 55 | KP86 | 16 | 1 | 2 |
| 56 | KP87 | 18 | 1 | 0 |
| 57 | KP88 | 15 | 1 | 3 |
| 58 | KP91 | 17 | 2 | 0 |
| 59 | KP92 | 16 | 2 | 1 |
| 60 | KP93 | 10 | 5 | 4 |
| 61 | KP94 | 17 | 0 | 2 |
| 62 | KP95 | 15 | 2 | 2 |
| 63 | KP96 | 17 | 0 | 2 |
| 64 | KP97 | 16 | 1 | 2 |
| 65 | KP98 | 18 | 0 | 1 |
| 66 | KP99 | 15 | 3 | 1 |
| 67 | KP100 | 16 | 1 | 2 |

**Table S5a.** The similarity indices of the isolates according to dendrogram 2.

| Isolate number | Distance | Similarity index percentage |
| --- | --- | --- |
| 49 | 3.3 | 93.4% |
| 46 | 3.3 | 93.4% |
| 83 | 5.2 | 89.6% |
| 61 | 10.5 | 79% |
| 40 | 18.0 | 64% |
| 63 | 5.6 | 88.8% |
| 56 | 5.6 | 88.8% |
| 55 | 11 | 77% |
| 35 | 10 | 80% |
| 18 | 10 | 80% |
| 15 | 3.8 | 92.4% |
| 14 | 3.8 | 92.4% |
| 12 | 14.9 | 70.2% |
| 10 | 18.9 | 62.2% |
| 16 | 11.1 | 77.8% |
| 11 | 11.1 | 77.8% |
| 7 | 15 | 70% |
| 3 | 20.7 | 58.6% |
| 2 | 27.7 | 44.6% |
| 9 | 13.6 | 72.8% |
| 8 | 13.6 | 72.8% |
| 5 | 20.2 | 59.6% |
| 91 | 10 | 80% |
| 87 | 10 | 80% |
| 85 | 15.4 | 69.2% |
| 93 | 43.1 | 13.8% |

**Table 5b.** The similarity indices of the isolates according to dendrogram 3.

| Isolate number | Distance | Similarity index % |
| --- | --- | --- |
| 75 | 16.7 | 66.6% |
| 76 | 16.7 | 66.6% |
| 73 | 18.4 | 63.2% |
| 84 | 21.9 | 56.2% |
| 82 | 7.9 | 84.2% |
| 77 | 7.9 | 84.2% |
| 81 | 8.9 | 82.2% |
| 80 | 7.9 | 84.2% |
| 79 | 7.9 | 84.2% |
| 95 | 6.3 | 87.4% |
| 92 | 6.3 | 87.4% |
| 97 | 13.9 | 72.2% |
| 88 | 11.1 | 77.8% |
| 86 | 11.1 | 77.8% |
| 96 | 21.5 | 57% |
| 94 | 27.9 | 44.2% |
| 99 | 23.3 | 53.4% |
| 98 | 23.3 | 53.4% |
| 71 | 29.1 | 41.8% |
| 100 | 40.1 | 19.8% |

**Table 5c.** The similarity indices of the isolates according to dendrogram 3.

| Isolate number | Distance | Similarity index percentage |
| --- | --- | --- |
| 50 | 13.6 | 72.8% |
| 43 | 13.6 | 72.8% |
| 39 | 10 | 80% |
| 13 | 10 | 80% |
| 24 | 16.7 | 66.6% |
| 6 | 16.7 | 66.6% |
| 33 | 10 | 80% |
| 30 | 10 | 80% |
| 36 | 15 | 70% |
| 29 | 12.5 | 75% |
| 20 | 12.5 | 75% |
| 21 | 18.8 | 62.4% |
| 66 | 10 | 80% |
| 59 | 10 | 80% |
| 17 | 22.7 | 54.6% |
| 69 | 12.5 | 75% |
| 67 | 12.5 | 75% |
| 70 | 18.8 | 62.4% |
| 58 | 11.5 | 77% |
| 52 | 11.5 | 77% |
| 64 | 26.4 | 47.2% |

**Table S6. Calculation of the mass concentration** **of AgNPs.**

Mp = π×10.49 × (19.67668 ×10^-7^)^3^/6

= 4.1844 ×10^-17^ gram

Np = (π×10.49 × (19.67668 ×10^-7^)3/ (6 × 107.8682)) × (6.02 ×10^23^)

= 233524 atom/NP

After that, the total number of Ag atoms in 100 ml of 0.0125 mM of AgNO3 was calculated.

Number of moles of Ag = number of moles of AgNO3 = 0.0125 M

N total = 0.0125 × 0.1 × (6.02 ×10^23^)

= 7.525 × 10^20^ atoms

Mtotal= (7.525 × 10^20^ × 4.1844 ×10^-17^) / 233524

= 0.1348 g

0.1348 g of AgNps dissolved in 100 ml(0.1 L) of deionized water

= 1.348 g/L

= 1.348 mg/ml

**Table S7.** The results of the grain size from each 2 theta and FWHM in XRD data

| 2 theta (2θ) | Theta (θ) | FWHM  In degree | FWHM  In radian | D of each peak (nm) |
| --- | --- | --- | --- | --- |
| 38.005 | 19.0025 | 0.549 | 0.009582 | 15.13445 |
| 44.489 | 22.2445 | 0.399 | 0.006964 | 21.2724 |
| 64.291 | 32.1455 | 0.549 | 0.009582 | 16.900549 |
| 77.26 | 38.63 | 0.898 | 0.015673 | 11.198696 |
| 81.5 | 40.75 | 0.349 | 0.00609 | 29.713733 |

**Table S8**. Inhibition zones of AgNPs, meropenem, and meropenem+ AgNPs for XDR *K. pneumoniae* isolates (n=29)

| **Serial Nr.** | **Isolate code** | **Average Inhibition zone (mm) ± SD** | | |
| --- | --- | --- | --- | --- |
|  |  | **AgNPs** | **Meropenem** | **Meropenem+ AgNPs** |
| 1 | KP2 | 22.0 ± 1.4 | 13.0 ± 1.4 | 28.0 ± 1.4 |
| 2 | KP5 | 16.5 ± 2.1 | 18.33 ± 1.5 | 29.0 ± 1.4 |
| 3 | KP12 | 16.0 ± 1.4 | 17.0 ± 0.0 | 22.5 ± 0.7 |
| 4 | KP16 | 15.33 ± 1.5 | 8.33 ± 1.5 | 22.0 ± 1.4 |
| 5 | KP36 | 22.0 ± 1.4 | 12.0 ± 0.0 | 26 .0± 1.4 |
| 6 | KP39 | 23.5 ± 2.1 | 11.67 ± 1.1 | 23.0 ± 1.4 |
| 7 | KP40 | 16.5 ± 2.1 | 10.5 ± 0.7 | 23.0 ± 1.4 |
| 8 | KP50 | 23.5 ± 2.1 | 10.0 ± 1.4 | 25.0 ± 0.0 |
| 9 | KP58 | 16.33 ± 1.1 | 10.67 ± 1.5 | 24.5 ± 0.7 |
| 10 | KP59 | 16.5 ± 2.1 | 10.67 ± 1.5 | 26.5 ± 2.1 |
| 11 | KP61 | 22.0 ± 1.4 | 10.5 ± 0.7 | 26.5 ± 2.1 |
| 12 | KP63 | 16.5 ± 2.1 | 10.5 ± 0.7 | 22 .0± 1.4 |
| 13 | KP64 | 17.0 ± 0.0 | 21.67 ± 1.5 | 26.5 ± 2.1 |
| 14 | KP71 | 16.5 ± 2.1 | 12.33 ± 1.5 | 26.5 ± 2.1 |
| 15 | KP73 | 16 .0± 1.4 | 15.67 ± 0.4 | 26.5 ± 2.1 |
| 16 | KP75 | 14.5 ± 0.7 | 10.0 ± 1.0 | 22.0 ± 1.4 |
| 17 | KP77 | 17.0 ± 0.0 | 22.5 ± 0.7 | 24.0 ± 1.4 |
| 18 | KP80 | 23.5 ± 2.1 | 18.5 ± 0.7 | 29.0 ± 1.41 |
| 19 | KP81 | 16.5 ± 2.1 | 24.5 ± 0.7 | 26.67 ± 1.5 |
| 20 | KP83 | 18.0 ± 0.0 | 9.5 ± 0.7 | 26.5 ± 2.1 |
| 21 | KP84 | 23.5 ± 2.1 | 10.5 ± 0.7 | 24.0 ± 1.4 |
| 22 | KP85 | 17.5 ± 0.7 | 10.0 ± 1.0 | 23.0 ± 1.4 |
| 23 | KP86 | 17.0 ± 1.4 | 12 .0± 1.0 | 26.5 ± 2.1 |
| 24 | KP93 | 14.5 ± 0.7 | 14 .0± 1.4 | 24.0 ± 1.4 |
| 25 | KP94 | 15.0 ± 0.0 | 13.0 ± 1.0 | 27.0 ± 1.4 |
| 26 | KP96 | 22.0 ± 1.4 | 17.0 ± 1.4 | 26.67 ± 1.5 |
| 27 | KP97 | 16.5 ± 2.1 | 11.0 ± 1.4 | 26.5 ± 2.1 |
| 28 | KP99 | 17.5 ± 0.7 | 10.5 ± 0.7 | 25.0 ± 0.0 |
| 29 | KP100 | 16.0 ± 1.4 | 13.0 ± 0.1 | 27.0 ± 0.0 |

**Table** **S9.** MIC and ΣFIC index values of AgNPs and meropenem for *K. pneumoniae* isolates

| **Nr.** | **Isolate code** | **MIC of AgNPs alone (mg/mL)** | **MIC of meropenem (µg/mL)** | **MIC of AgNPs in combination (mg/mL)** | **MIC of meropenem in combination (µg/mL)** | **ΣFIC index** |
| --- | --- | --- | --- | --- | --- | --- |
| 1 | K2 | 0.674 | 12.5 | 0.1685 | 3.125 | 0.5(S) |
| 2 | K5 | 0.337 | 12.5 | 0.1685 | 3.125 | 0.75(Ps) |
| 3 | K12 | 0.337 | 12.5 | 0.1685 | 3.125 | 0.75(Ps) |
| 4 | K16 | 0.674 | 12.5 | 0.1685 | 3.125 | 0.5(S) |
| 5 | K36 | 0.337 | 12.5 | 0.08425 | 3.125 | 0.5(S) |
| 6 | K39 | 0.337 | 12.5 | 0.08425 | 3.125 | 0.5(S) |
| 7 | K40 | 0.674 | 12.5 | 0.08425 | 3.125 | 0.375(S) |
| 8 | K50 | 0.674 | 12.5 | 0.08425 | 1.5625 | 0.25(S) |
| 9 | K58 | 0.674 | 12.5 | 0.08425 | 3.125 | 0.375(S) |
| 10 | K59 | 0.674 | 12.5 | 0.08425 | 1.5625 | 0.25(S) |
| 11 | K61 | 0.674 | 12.5 | 0.08425 | 3.125 | 0.375(S) |
| 12 | K63 | 0.337 | 12.5 | 0.08425 | 3.125 | 0.375(S) |
| 13 | K64 | 0.674 | 12.5 | 0.1685 | 1.5625 | 0.375(S) |
| 14 | K71 | 0.337 | 12.5 | 0.1685 | 3.125 | 0.75(Ps) |
| 15 | K73 | 0.674 | 12.5 | 0.1685 | 3.125 | 0.5(S) |
| 16 | K75 | 0.337 | 12.5 | 0.08425 | 3.125 | 0.5(S) |
| 17 | K77 | 0.337 | 12.5 | 0.1685 | 3.125 | 0.75(Ps) |
| 18 | K80 | 0.337 | 12.5 | 0.1685 | 3.125 | 0.75(Ps) |
| 19 | K81 | 0.337 | 12.5 | 0.1685 | 3.125 | 0.75(Ps) |
| 20 | K83 | 0.674 | 12.5 | 0.08425 | 1.5625 | 0.25(S) |
| 21 | K84 | 0.674 | 12.5 | 0.08425 | 3.125 | 0.375(S) |
| 22 | K85 | 0.337 | 12.5 | 0.1685 | 3.125 | 0.75(Ps) |
| 23 | K86 | 0.337 | 12.5 | 0.1685 | 3.125 | 0.75(Ps) |
| 24 | K93 | 0.337 | 12.5 | 0.08425 | 1.5625 | 0.375(S) |
| 25 | K94 | 0.674 | 12.5 | 0.08425 | 3.125 | 0.375(S) |
| 26 | K96 | 0.674 | 12.5 | 0.1685 | 6.25 | 0.75(Ps) |
| 27 | K97 | 0.337 | 12.5 | 0.1685 | 3.125 | 0.75(Ps) |
| 28 | K99 | 0.674 | 12.5 | 0.1685 | 3.125 | 0.5(S) |
| 29 | K100 | 0.674 | 12.5 | 0.08425 | 3.125 | 0.375(S) |

AgNPs, silver nanoparticles; MIC, minimum inhibitory concentration; ΣFIC index, sum of fraction inhibitory index; S (synergy); Ps, partial Synergy.


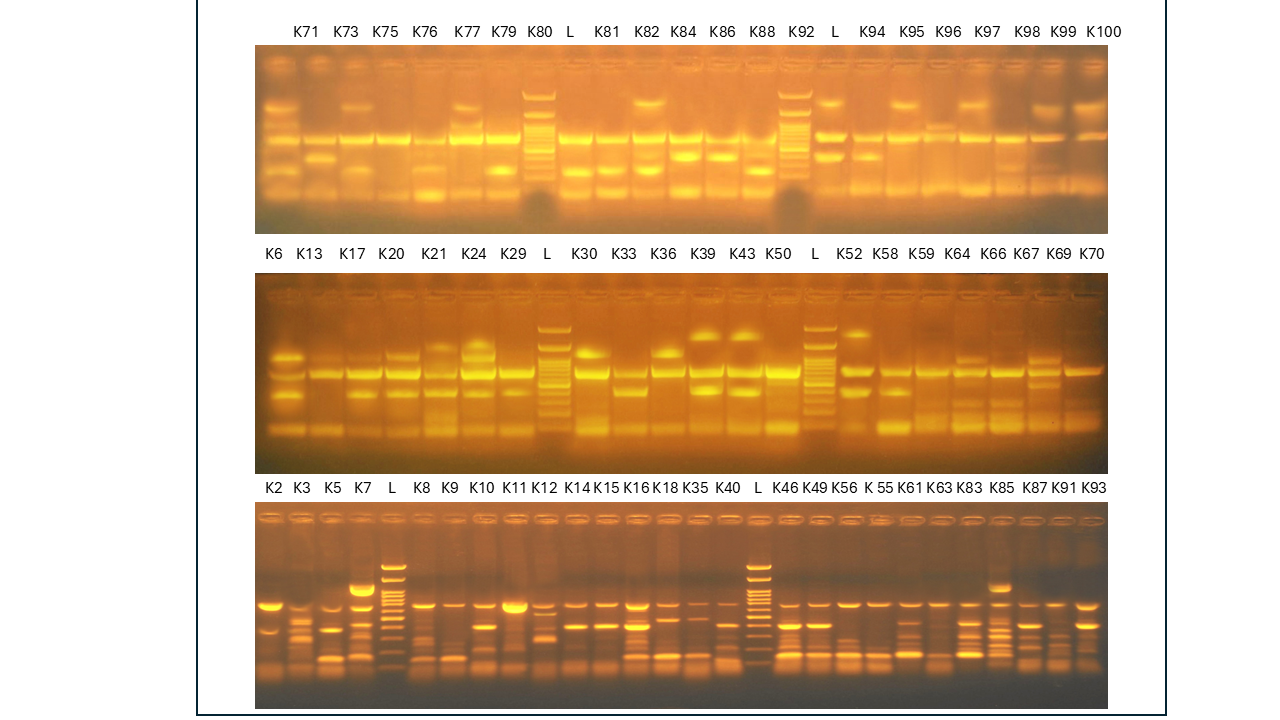


**Figure S1.** Gel electrophoresis for the 67 isolates of *Klebsiella pneumoniae* after ERIC-PCR
